# Integrated dual transcriptome sequencing and experimental validation reveal potential mechanisms of baicalin against *pneumocystis* pneumonia in immunosuppressed rats

**DOI:** 10.3389/fmicb.2026.1923465

**Published:** 2026-08-26

**Authors:** Ting Xue, Lijuan Fan, Weiqin Du, Xingxu Chen, Chaofeng Liu, Wenjuan Dai, Jia Xu, Lufeng Chen, Shouan Ren

**Affiliations:** 1 State Key Laboratory for Pneumoconiosis of National Health Commission, First Hospital of Shanxi Medical University, Taiyuan, Shanxi, China; 2 Key Laboratory of Prevention and Treatment of Chronic Respiratory Diseases and Pneumoconiosis in Shanxi Province, First Hospital of Shanxi Medical University, Taiyuan, Shanxi, China; 3 Department of Respiratory and Critical Care Medicine, First Hospital of Shanxi Medical University, Taiyuan, Shanxi, China; 4 Department of Clinical Laboratory, The First People’s Hospital of Lvliang, Lvliang, Shanxi, China; 5 Department of Radiation Oncology, First Hospital of Shanxi Medical University, Taiyuan, Shanxi, China; 6 Clinical Practice Teaching Center, Shenyang Medical College, Shenyang, Liaoning, China

**Keywords:** baicalin, dual RNA-Seq, mechanism, Nrf2, *PcRtt109*, *Pneumocystis* pneumonia

## Abstract

**Background:**

*Pneumocystis* pneumonia (PCP) remains a major cause of morbidity and mortality in immunocompromised individuals. Although baicalin (Ba), a natural bioactive flavonoid, has demonstrated protective and therapeutic effects against PCP, its molecular mechanisms remain undefined. We employed dual RNA sequencing (dual RNA-seq) to characterize host and pathogen transcriptional responses to Ba treatment in an immunosuppressed rat model of PCP.

**Methods:**

Comparative transcriptomic analyses identified differentially expressed genes in both the host and *Pneumocystis*, followed by Gene Ontology, Kyoto Encyclopedia of Genes and Genomes, and gene set enrichment analyses. Candidate targets were further investigated using network pharmacology, protein–protein interaction analysis, molecular docking, and molecular dynamics simulations. Key findings were validated by immunohistochemistry, enzyme-linked immunosorbent assay, and quantitative PCR.

**Results:**

Ba markedly remodeled host and pathogen transcriptomes. Host transcriptomic analyses showed that Ba attenuated inflammatory and oxidative stress responses by modulating immune-related pathways, including Toll-like receptor, NF-κB, cytokine-cytokine receptor interaction, chemokine signaling, Th17 cell differentiation, and antigen processing and presentation. Experimental validation demonstrated that Ba reduced pulmonary expression of indoleamine 2,3-dioxygenase 1 (IDO1), Toll-like receptor 2 (TLR2), and TLR4 while increasing nuclear factor erythroid 2-related factor 2 (Nrf2) and its downstream antioxidant enzyme heme oxygenase-1 (HO-1). Pathogen transcriptomic analysis identified *Pneumocystis Rtt109* (*PcRtt109*), a fungal histone acetyltransferase, as a potential pathogen-specific target that was significantly downregulated after Ba treatment. Molecular docking and molecular dynamics simulations supported stable interactions between Ba and IDO1, Nrf2, TLR2, TLR4, and *PcRtt109*, with the strongest predicted binding observed for *PcRtt109*.

**Conclusion:**

Dual RNA-seq revealed that Ba exerts anti-PCP activity through coordinated modulation of host and pathogen molecular networks. Its therapeutic effects are associated with suppression of inflammatory signaling, enhancement of antioxidant defenses, and inhibition of a fungal virulence-associated target. These findings provide mechanistic insights into host-pathogen interactions during PCP and support Ba as a potential therapeutic candidate for PCP.

## Graphical abstract

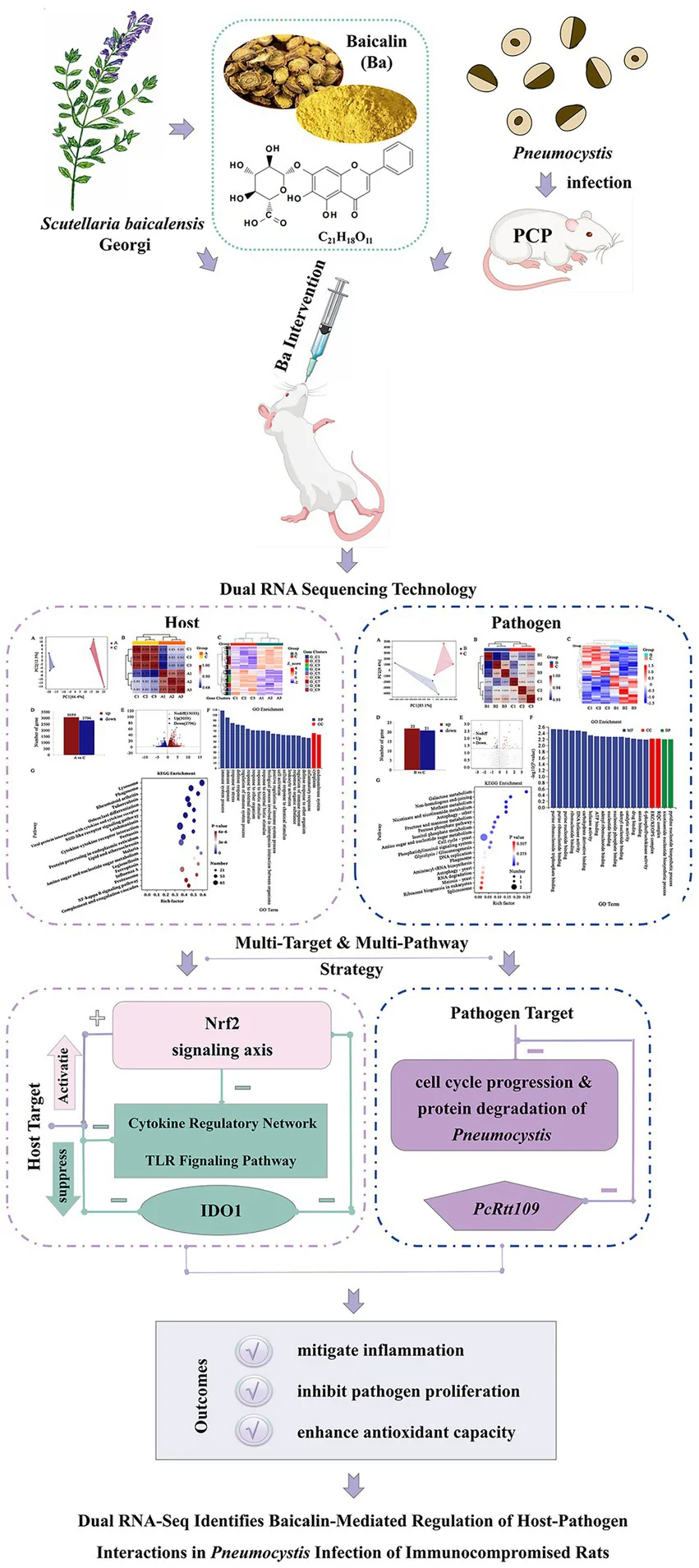

## 1 Introduction

*Pneumocystis* pneumonia (PCP) is a severe and potentially life-threatening condition that primarily affects immunocompromised individuals, particularly those living with human immunodeficiency virus (HIV). Although the introduction of highly active antiretroviral therapy (HAART) and PCP prophylaxis has significantly reduced the incidence of HIV-associated PCP (Lopez-Sanchez et al., 2015; Huang et al., 2017), this infection remains the most common opportunistic complication in HIV-positive patients. In recent years, the prevalence of PCP among HIV-negative immunocompromised individuals has risen steadily, with such cases exhibiting more abrupt clinical onset, poorer prognosis, and higher mortality rates compared to their HIV-positive counterparts (Avino et al., 2016; Salzer et al., 2018; Alsayed et al., 2022; Xue et al., 2023).

Trimethoprim-sulfamethoxazole (TMP-SMX), the standard drug for PCP prevention and treatment, has contributed to reducing disease incidence. However, its widespread use has raised concerns over adverse side effects, such as hypersensitivity reactions, and the emergence of TMP-SMX-resistant *Pneumocystis* strains. Mutations in dihydropteroate synthase (*dhps*), associated with TMP-SMX resistance, have been implicated in poor clinical outcomes among PCP patients (Helweg-Larsen et al., 1999; de la Horra et al., 2021). Compounding these challenges, the inability to culture *Pneumocystis jirovecii* (the human-specific *Pneumocystis* species) in standardized systems has hindered routine drug susceptibility testing and resistance detection (Ma et al., 2018). Thus, the development of novel, effective therapeutic strategies against PCP is of paramount importance for improving patient outcomes.

Baicalin (Ba), a bioactive flavone derived from the traditional Chinese herb Scutellaria baicalensis, has garnered attention for its diverse pharmacological activities, including anti-inflammatory, antiviral, antitumor, antibacterial, antioxidant, and neuroprotective properties (Wen et al., 2023). Our recent studies indicated that Ba exhibits both therapeutic and preventive effects against *Pneumocystis* infection in immunosuppressed rats. These effects are mediated through anti-inflammatory and antioxidant activities, regulation of protease/anti-protease balance, and attenuation of pulmonary collagen deposition (Xue et al., 2025). However, the molecular mechanisms underpinning these effects and the precise therapeutic targets of Ba remain unclear.

RNA sequencing (RNA-Seq), a transformative next-generation sequencing (NGS) technology, has emerged as a vital tool for quantitative transcriptome analysis, enabling robust comparisons between diseased and healthy tissues (Goodwin et al., 2016; Dominguez et al., 2017; Yang et al., 2020). By offering unparalleled sensitivity and specificity, RNA-Seq has become pivotal in elucidating disease mechanisms, identifying therapeutic targets, and evaluating drug mechanisms (Wang et al., 2009; Goodwin et al., 2016; Sun and Hu, 2016; Yang et al., 2020; de Jong and Bosco, 2021; Smail and Montgomery, 2024). Notably, dual RNA-Seq extends the capabilities of RNA-Seq by simultaneously capturing global transcriptomic changes in both a host and its associated pathogen during infection (Westermann et al., 2012; Westermann et al., 2017; Westermann and Vogel, 2021). This technique allows for an integrated exploration of host-pathogen interactions, providing insights into the molecular networks governing virulence, pathology, and therapeutic interventions. Dual RNA-Seq has proven invaluable in studying complex host-pathogen dynamics and guiding targeted therapeutic strategies for microbial infections (Westermann et al., 2012; Westermann et al., 2017; Westermann and Vogel, 2021).

Building upon our previous findings on Ba′s protective effects against *Pneumocystis* infection (Xue et al., 2025), the current study employed dual RNA-Seq to further explore the molecular mechanisms underlying these effects. By focusing on the interactions between host and pathogen, we identified the pathways and potential therapeutic targets modulated by Ba in PCP. This research contributes to a deeper understanding of *Pneumocystis* pathophysiology and provides novel insights into the clinical application of Ba for PCP treatment and prevention, offering a foundation for the development of innovative therapeutic strategies.

## 2 Materials and methods

### 2.1 Animal model establishment and lung tissue processing

The study protocol for animal experimentation was approved by the Animal Ethics Committee of Shanxi Medical University (Approval No. DWLL-2024-018). The animal model of PCP was established in female Wistar rats via immunosuppression induced by intraperitoneal injection of dexamethasone sodium phosphate (3 mg, twice weekly) over 12 weeks, as detailed in our previous study (Xue et al., 2025). Briefly, to evaluate both the prophylactic and therapeutic effects of Ba, rats were randomly assigned to eight groups (*n* = 8/group), including immunocompetent controls, untreated PCP model, Ba-normal, Ba-prevention, Ba-low/medium/high-dose therapeutic, and a positive control (TMP-SMZ-treated) group. After 8 weeks, one rat per group was sacrificed under anesthesia for *Pneumocystis* identification. The key interventions are summarized as follows: Ba was administered intragastrically once daily at doses of 100, 200, or 400 mg/kg for therapeutic evaluation starting at week 8 post-immunosuppression, while the prophylactic regimen (100 mg/kg) commenced at week 5. The TMP-SMZ group received equivalent treatment from week 8. All immunosuppressed rats continued dexamethasone until the study endpoint (*n* = 7/group). Detailed procedures regarding animal housing, monitoring, and *Pneumocystis* identification have been described previously (Xue et al., 2025).

### 2.2 RNA extraction, library preparation, and sequencing

Total RNA in lung tissues randomly sampled from the control group, middle-dose group, and PCP untreated group (*n* = 3 per group) were extracted using the RNAiso Plus reagent (Takara Bio, Dalian, China). RNA extracts were submitted for RNA-Seq by commercial sequencing services (Personal Biotechnology, Shanghai, China). This sample size was selected to balance the statistical power needed for detecting large-fold-change genes with the ethical imperatives of animal research in this exploratory study. RNA integrity and concentration were assessed using multiple methods to ensure quality: purity (A260/A280 and A260/A230 ratios) was measured with a NanoDrop 2000 spectrophotometer (Thermo Scientific, United States); RNA Integrity Number (RIN) was determined using an Agilent 2,100 Bioanalyzer with the RNA 6000 Nano Kit (Agilent Technologies, United States). Strand-specific RNA-seq libraries were prepared using the NEBNext Ultra Directional RNA Library Prep Kit for Illumina (New England Biolabs, United States) following the manufacturer’s instructions. Briefly, poly(A) + mRNA was enriched from total RNA (≥1 μg) using oligo(dT) magnetic beads. The enriched mRNA was then fragmented using divalent cations under elevated temperature. First-strand cDNA was synthesized using random oligonucleotides and reverse transcriptase, followed by second-strand cDNA synthesis. The double-stranded cDNA was purified, end-repaired, adenylated at the 3′ ends, and ligated to Illumina sequencing adaptors. cDNA fragments of approximately 400–500 bp were selected using AMPure XP beads (Beckman Coulter, United States). The selected fragments were enriched by PCR amplification and purified again with AMPure XP beads to generate the final cDNA libraries. The quality and concentration of the constructed libraries were rigorously quantified. Library fragment size distribution was assessed using an Agilent 2,100 Bioanalyzer with the High Sensitivity DNA Kit (Agilent Technologies, United States). The total library concentration was measured with the Quant-iT PicoGreen dsDNA Assay Kit (Invitrogen, United States) on a Quantifluor-ST fluorometer (Promega, United States). The effective library concentration was accurately determined by quantitative PCR (qPCR) on a StepOnePlus Real-Time PCR System (Thermo Scientific, United States) to ensure precise clustering during sequencing. Multiplexed libraries were pooled in equimolar ratios based on the qPCR results. The pool was sequenced on an Illumina NovaSeq 6,000 platform (Illumina, United States) using a 150-base paired-end read strategy, generating an average of over 20 million raw read pairs per library. All raw reads were subsequently subjected to a series of stringent quality control steps.

Data Quality Control and Filtering: Raw sequencing data (FASTQ files) were subjected to stringent quality control. Adapters and low-quality reads were trimmed using fastp (v0.22.0) with the following parameters: removal of reads containing adapter sequences and discarding reads with an average quality score below Q20. The resulting high-quality data, referred to as “clean reads,” were used for all downstream analyses.

Read Mapping and Quantification: To simultaneously analyze host and pathogen transcriptomes, a dual-reference genome approach was employed. Clean reads from each sample were independently aligned to the host-pathogen respective reference genomes using HISAT2 (v2.1.0). Indexes for the host and pathogen reference genomes were built using HISAT2-build. Ambiguously mapped reads that did not satisfy the mapping criteria were excluded according to the default settings of the alignment software. Gene-level read counts were then generated using HTSeq (v0.9.1) in union mode, producing raw count matrices for host and pathogen genes separately.

### 2.3 Dual RNA-Seq analysis

The analysis of differentially expressed genes (DEGs), Gene Ontology (GO) enrichment and Kyoto Encyclopedia of Genes and Genomes (KEGG) enrichment were, respectively, using software including DESeq 1.38.3 version, topGO 2.50.0 version, and clusterProfiler 4.6.0 version. These raw counts served as the direct input for the initial genome-wide differential expression analysis, which was performed with DESeq2 (v1.38.3), using a significance threshold of |log2FoldChange| > 1 and a *p* < 0.05. Benjamini-Hochberg false discovery rate (FDR) correction was applied. Separately, FPKM (Fragments Per Kilobase of transcript per Million mapped fragments) values were calculated only for the purpose of gene expression visualization and inter-sample comparisons, as this normalized value is more intuitive for assessing relative expression levels.

**Figure 1:**
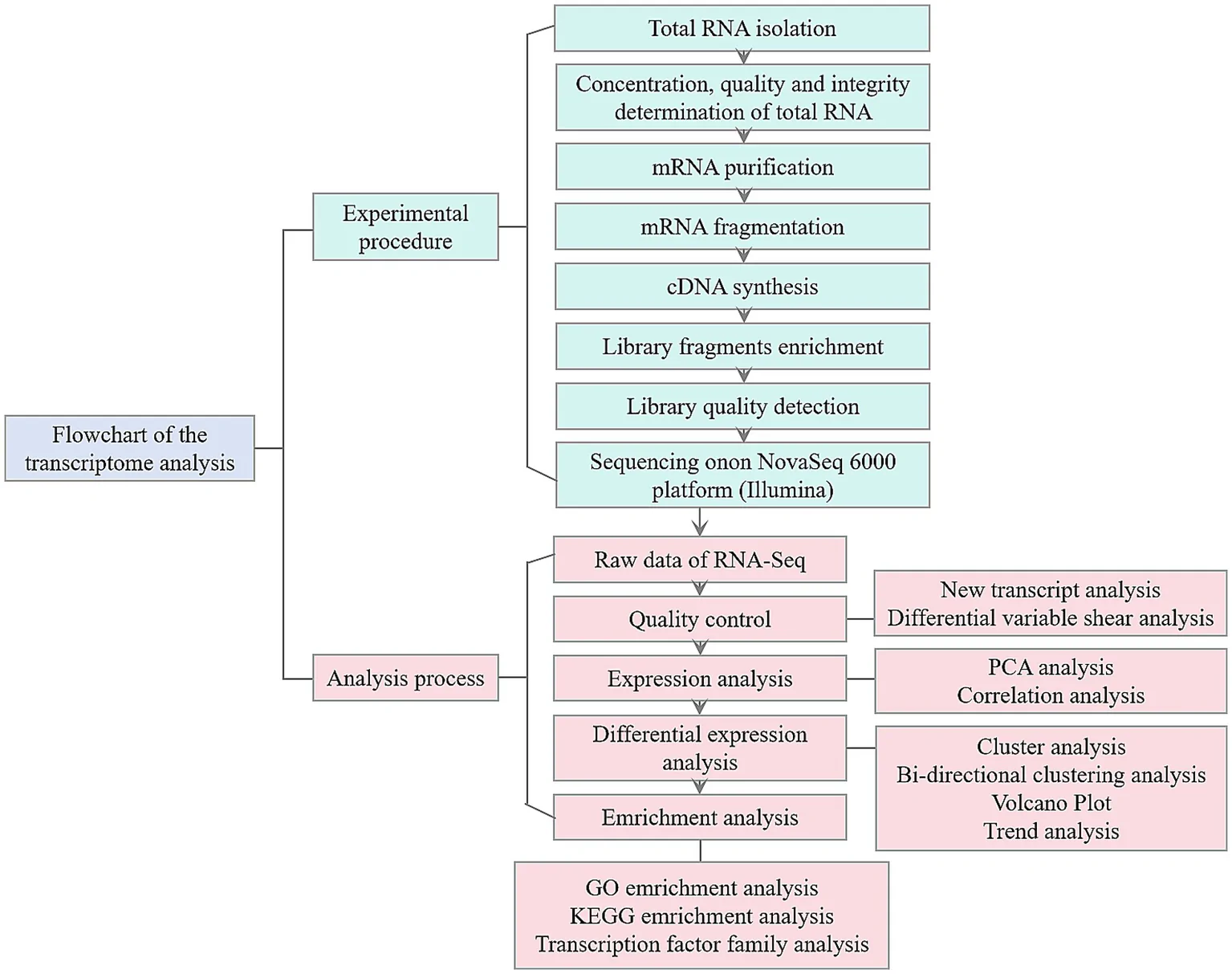
Flowchart of the transcriptome analysis. PCA, principal component analysis; GO, gene ontology; KEGG, kyoto encyclopedia of genes and genomes.

At the same time, we used R language Pheatmap (v1.0.12) software package to perform bi-directional clustering analysis of all different genes of samples. We got heatmap according to the expression level of the same gene in different samples and the expression patterns of different genes in the same sample with Euclidean method to calculate the distance and Complete Linkage method to cluster. Enrichment analysis: we mapped all the genes to Terms in the Gene Ontology database and calculated the numbers of differentially enriched genes in each Term. Using top GO (v2.50.0) to perform GO enrichment analysis on the differential genes (all DEGs/up DEGs/down DEGs), calculate *p*-value by hypergeometric distribution method (the standard of significant enrichment is *p* < 0.05), and find the GO term with significantly enriched differential genes to determine the main biological functions performed by differential genes. ClusterProfiler (v4.6.0) software was used to carry out the enrichment analysis of the KEGG pathway of differential genes, focusing on the significant enrichment pathway with *p* < 0.05. Gene set enrichment analysis (GSEA) was performed on the entire gene list using the clusterProfiler package (v4.6.0) to evaluate coordinated expression changes in predefined biological pathways. All genes were ranked based on the log2 Fold Change (log2FC) metric derived from the comparison between experimental groups. Gene sets were constructed based on the GO and KEGG annotations obtained in our transcriptomic analysis pipeline. The statistical significance of the enrichment was determined by calculating the Normalized Enrichment Score (NES) and the corresponding False Discovery Rate (FDR) *q*-value. In accordance with common practice and the clusterProfiler framework, gene sets with |NES| > 1, a nominal *p* < 0.05, and an FDR *q* < 0.25 were considered statistically significant. Flowchart of the transcriptome analysis is shown in Figure 1.

The reference genome of the above analysis we used is the genome of *Rattus norvegicus*.^1^ The screened conditions used for analyzing were as follows: to reduce false positives in subsequent integrative analyses (e.g., network pharmacology and transcriptomic overlap), we identified DEGs using a more stringent threshold of |log2FoldChange| > 1.2 (adjusted *p* < 0.05) to prioritize high-confidence candidates; to focus on the most robust candidate targets, a final, more stringent screening was applied using a cutoff of |log2FoldChange| > 2 (adjusted *p* < 0.05). Then, we further analyzed the DEGs, GO enrichment and KEGG enrichment using the reference genome of *Pneumocystis carinii* B80 (Ma et al., 2016),^2^ based on two threshold parameters, namely, expression difference multiple |log2FoldChange| > 1.2 and significant *p* < 0.05.

### 2.4 Reverse transcription-quantitative polymerase chain reaction

Total RNA was extracted from lung tissues using RNAiso Plus reagent (Takara Bio, Dalian, China). RNA concentrations were quantified with a NanoDrop One C UV–Vis spectrophotometer (Thermo Scientific™), and each sample was diluted to a final concentration of 1 μg RNA. Reverse transcription was performed using the PrimeScript™ RT reagent kit with gDNA Eraser (Takara Bio, Dalian, China) following the manufacturer’s instructions. Quantitative PCR (qPCR) was conducted on the ABI 7500 real-time PCR instrument (Thermo Fisher Scientific, Inc.) using the TB Green^®^ Premix Ex Taq™ II (Tli RNase H Plus; Takara Bio, Dalian, China) kit in 20 μL reaction volumes.

Thermocycling conditions for qPCR amplification were as follows: Initial denaturation at 95 °C for 30 s, followed by 40 cycles of 95 °C for 5 s and 60 °C for 34 s. Primers specific for *Pneumocystis carinii Rtt109* (*PcRtt109*) were adopted from previously published sequences (Kottom et al., 2011). Primer sequences for GAPDH, used as the internal control, were as follows: forward, 5′-GACATGCCGCCTGGAGAAAC-3′; reverse, 5′-AGCCCAGGATGCCCTTTAGT-3′. Data analysis was conducted using the 2^−ΔΔCq method (Livak and Schmittgen, 2001) to normalize *PcRtt109* expression against GAPDH. Each experiment was performed with three biological replicates (*n* = 7 rats per group) and technical duplicates to ensure accuracy and reproducibility.

### 2.5 Immunohistochemistry

IHC was carried out using the streptavidin-peroxidase method and the UltraSensitive™ SP IHC kit (Maixin Biotech, Fuzhou, China) following the manufacturer’s guidelines. Paraffin-embedded lung tissue (*n* = 7 rats per group) sections were deparaffinized and subjected to antigen retrieval using sodium citrate buffer (pH 6.0). Subsequently, the sections were blocked with goat serum for 30 min at 37 °C and incubated with primary antibodies targeting nuclear factor erythroid 2-related factor 2 (Nrf2, Cat No. 16396-1-AP; dilution 1:300; Proteintech, Wuhan, China), indoleamine 2,3-dioxygenase 1 (IDO1, Cat No. DF6502; dilution 1:100; Affinity Biosciences, Wuhan, China), toll-like receptor (TLR4, Cat No. bs-1021R; dilution 1:200; Bioss Biosciences, Beijing, China), and TLR2 (Cat No. A11225; dilution 1:100; ABclonal Biotechnology, Wuhan, China) overnight at 4 °C. Following PBS washes, sections were incubated with the streptavidin-peroxidase complex (37 °C, 30 min) and then developed with a DAB substrate kit (cat no. 0031; Maixin Biotech, Fuzhou, China) (cat no. 0031; Maixin Biotech, Fuzhou, China). Following counterstaining and mounting, slides were examined under a light microscope. Three fields of view exhibiting positive brown staining (DAB signal) were randomly selected per slide and digitally captured. To ensure objective quantification, image analysis was performed in a blinded manner. The integrated optical density (IOD) of the DAB signal within these fields was measured semi-automatically using Image-Pro Plus 6.0 software (Media Cybernetics, Rockville, MD, United States).

### 2.6 Enzyme-linked immunosorbent assay (ELISA)

The serum levels of Nrf2 (NFE2L2) and heme oxygenase-1 (HO-1) in rats (*n* = 7/group) were quantified using a commercial double-antibody sandwich ELISA kit (Rat NFE2L2 ELISA Kit, Cat No. E-EL-R1052; Rat HO-1 ELISA Kit, Cat No. E-EL-R0488; Elabscience Biotechnology, Wuhan, China), following the manufacturer’s instructions. Briefly, captured antibodies bound to NFE2L2 and HO-1 in the samples and standards. After incubation with a biotinylated detection antibody and horseradish peroxidase (HRP)-conjugated avidin, the resulting immune complexes were washed. The reaction was developed with tetramethylbenzidine (TMB) substrate, stopped with stop solution, and the absorbance was measured at 450 nm. The Nrf2 and HO-1 concentration in each sample was determined by interpolating from the standard curve.

### 2.7 Identification of Ba-PCP common targets

Collection of Ba Targets: Prediction of Ba targets was conducted using its canonical SMILES structure from PubChem as the query for the following databases: Swiss Target Prediction, STITCH, and the SEA Search Server.

Prediction of PCP-Related Targets: To comprehensively identify targets associated with PCP, a search was performed using the keyword “*Pneumocystis* pneumonia” across five public databases: Genecards, OMIM, NCBI, Open Targets Platform, and the Comparative Toxicogenomics Database (CTD). The target lists obtained from each database were merged, and duplicates were removed to generate a non-redundant set of PCP-related genes for subsequent analysis.

Acquisition of Common Targets: The intersection between the potential targets of Ba and the PCP-related candidate gene set was determined using the online Venn platform^3^ to define the predicted drug-disease targets.

### 2.8 Screening of key targets and network pharmacology analysis

The overlapping potential target genes or DEGs obtained from various datasets for subsequent analysis were integrated and analyzed using the Venny 2.1 online tool.^4^ Then identified via the Venn diagram. Protein–protein interaction (PPI) networks were constructed using the STRING database^5^ to explore functional associations between the identified protein targets. PPI analyses were performed under standard parameters to assess molecular interactions, which provide a comprehensive framework for understanding the biological networks involved in Ba′s therapeutic mechanisms.

### 2.9 Molecular docking

To investigate the interactions between Ba and potential targets, molecular docking analysis was performed. The canonical three-dimensional (3D) structure of Ba (CAS No. 606–86-1) was retrieved from the PubChem database.^6^ Protein structures were obtained from the UniProt database^7^ using their respective entry identifiers.

Protein structures were prepared for docking by removing existing ligands and water molecules, adding hydrogen atoms, optimizing amino acid residues, and calculating charges using AutoDock Tools (ADT) v1.5.6. For ligand preparation, protonation and optimization of the Ba structure were also conducted using ADT v1.5.6. The ligand’s 2D structure was illustrated using ChemDraw 19.0 software, while its 3D structure was obtained from PubChem. A grid box was generated using the receptor grid generation wizard to define the docking region.

Molecular docking analysis was implemented using AutoDock Vina v1.1.2 and the GLIDE program. The PDB protein structures were converted to PDBQT format for docking, and the docking results were visualized using Discovery Studio 2019 Client. AlphaFold was used to validate the protein structures, with per-residue confidence scores (pLDDT) to identify regions of higher or lower structural confidence. In cases of low pLDDT values, adjustments based on unstructured regions were performed as necessary.

Binding affinities between Ba and the target proteins were evaluated based on docking scores computed using scoring functions. Docking poses were ranked by binding energy, and the pose with the best score (lowest binding energy) was selected for further analysis. Binding energies for optimal poses demonstrated strong binding affinity: scores < −7.0 kcal/mol indicated the best binding energy, scores < −5.0 kcal/mol suggested moderate binding affinity, and scores near −4.6 kcal/mol reflected basic interactions.

### 2.10 Molecular dynamics simulations for assessment of binding stability

To evaluate the binding stability of the Ba-candidate target complexes identified by molecular docking, molecular dynamics simulations (100 ns) were performed using GROMACS 2025 (Van Der Spoel et al., 2005). The AMBER14SB force field was applied to the protein, while the General AMBER Force Field (GAFF2) was used to generate the topology parameters for the ligand (Krieger and Vriend, 2015; Patel et al., 2020). The topology files of the protein and ligand were merged with consistency in atom types ensured. The complex was solvated in a cubic periodic boundary box with a minimum distance of 1.2 nm between the complex and the box edges, using the TIP3P water model. Na^+^ and Cl^−^ ions were added to neutralize the system and to maintain an ionic concentration of 0.15 M, mimicking a physiological environment. The system underwent energy minimization followed by a two-step equilibration protocol: first, under the NVT ensemble for 2 ns, and then under the NPT ensemble for another 2 ns, each with a coupling constant of 0.1 ps. Production MD simulation was conducted for 100 ns (50,000,000 steps, 2 fs per step) under constant temperature (310 K) and pressure (1 bar), with coordinates saved every 1,000 steps for subsequent analysis. The binding free energy was calculated using the Molecular Mechanics Poisson-Boltzmann Surface Area (MMPBSA) method on frames extracted from the stable phase of the simulation.

### 2.11 Statistical analysis

Statistical analyses were performed using SPSS software (version 13.0) and GraphPad Prism software (version 8). Quantitative data conforming to a normal distribution are presented as mean ± standard deviation (SD). normality was assessed using the Shapiro–Wilk test, and homogeneity of variance was evaluated using levene’s test. For comparisons among multiple groups, one-way anova was performed. When the assumption of equal variances was met, Tukey’s *post-hoc* test was used; otherwise, the Games-Howell *post-hoc* test was applied. For comparisons between two groups, Student’s *t*-test was used for normally distributed data, whereas the Mann–Whitney U test was used for non-normally distributed data. A *p* < 0.05 was considered statistically significant. The selected *post-hoc* tests (Tukey and Games-Howell) account for multiple pairwise comparisons by controlling the family-wise error rate.

## 3 Results

### 3.1 Dual RNA-Seq analysis

To investigate the underlying mechanisms and potential targets involved in Ba′s therapeutic effects on PCP, dual RNA-Seq was performed on RNA samples obtained from three rats per group, including the control group (Group A), the Ba-middle-dose group (Group B), and the PCP untreated group (Group C). Samples were collected at the end of the animal model establishment phase. A detailed summary of the sequencing quality metrics, including total clean reads, Q20/Q30 values, mapping statistics, and consistency across replicates, is provided in Supplementary material 1.

**Figure 2:**
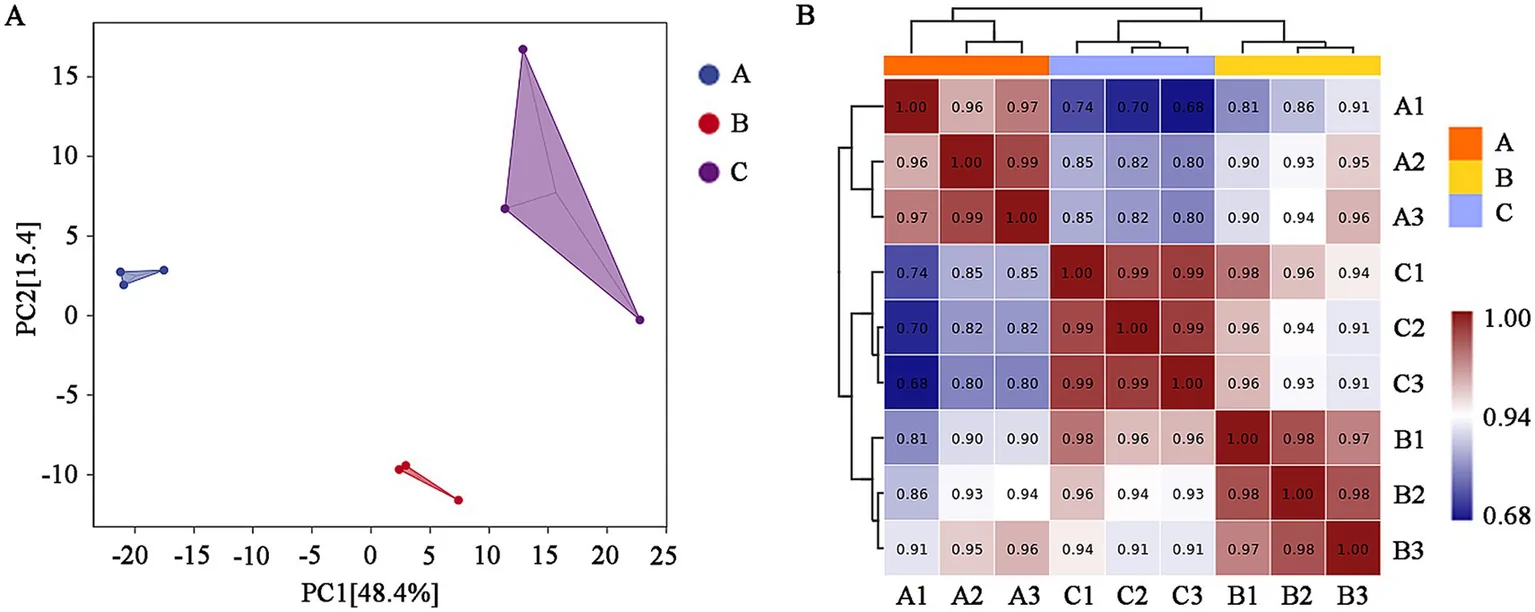
Transcriptomic profiling reveals inter-group and intra-group relationships among experimental conditions (*n* = 3/group). **(A)** PCA of global gene expression. The scatter plot illustrates the separation and clustering of samples from the Control, untreated PCP, and Ba-medium groups based on the first two principal components (PC1 and PC2). The percentage of variance explained by each component is indicated. Convex hulls are drawn to highlight group-wise clustering, demonstrating the transcriptional distinctions between conditions. **(B)** Inter-sample correlation heatmap. The heatmap displays Pearson (or Spearman) correlation coefficients calculated from the gene expression profiles of all samples. Hierarchical clustering is applied to both rows and columns. The color scale represents the degree of correlation, with darker colors indicating higher similarity. This analysis visually assesses the consistency within replicates of the same group and the divergence between different experimental groups. PCA, principal component analysis; Ba, baicalin; PCP, *Pneumocystis* pneumonia; A, control group; B, Ba-middle-dose group; C, untreated PCP group.

To assess the overall transcriptional changes, we performed an unsupervised principal component analysis (PCA) on the transcriptome data from the Control, untreated PCP, and Ba-middle-dose-treated groups. The PCA plot (Figure 2A) showed a clear separation among the three groups, with replicate samples clustering closely within each group. The untreated PCP group was distinctly separated from the Control group along the first principal component (PC1, 48.4% variance). The Ba-treated group exhibited a profile positioned between the Control and untreated PCP groups. To evaluate reproducibility and inter-group similarity, a sample-to-sample correlation analysis was performed based on global gene expression profiles. The resulting hierarchical clustering heatmap of Pearson correlation coefficients (Figure 2B) showed high correlation coefficients (PCC > 0.97) among replicates within each group. The three experimental groups formed distinct clusters.

**Figure 3:**
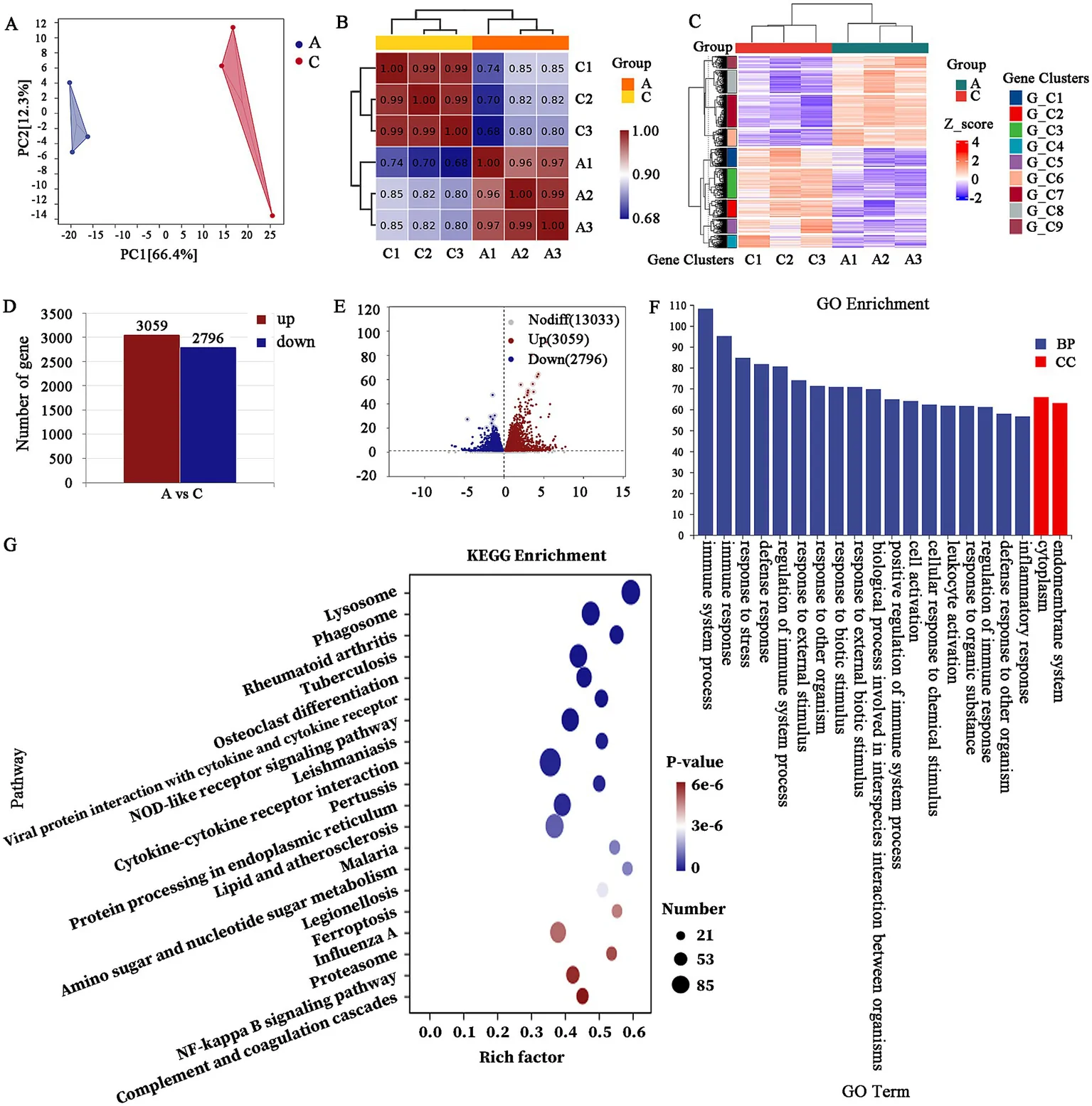
Transcriptome analysis of RNA-Seq data from control group vs. PCP untreated group (*n* = 3/group), using the reference genome of *Rattus norvegicus* (http://asia.ensembl.org/Rattus_norvegicus/Info/Index?db=core), including PCA plot **(A)**, sample correlation heatmap **(B)**, heatmap **(C)**, statistics of DEGs **(D)**, volcano plot **(E)**, bubble chart of GO enrichment analyses **(F)**, and bar plot of KEGG enrichment analyses **(G)**. A, control group; C, untreated PCP group; Ba, Baicalin; PCP, *Pneumocystis* pneumonia; PCA, Principal component analysis; DEGs, differentially expressed genes; GO, gene ontology; KEGG, kyoto encyclopedia of genes and genomes.

**Figure 4:**
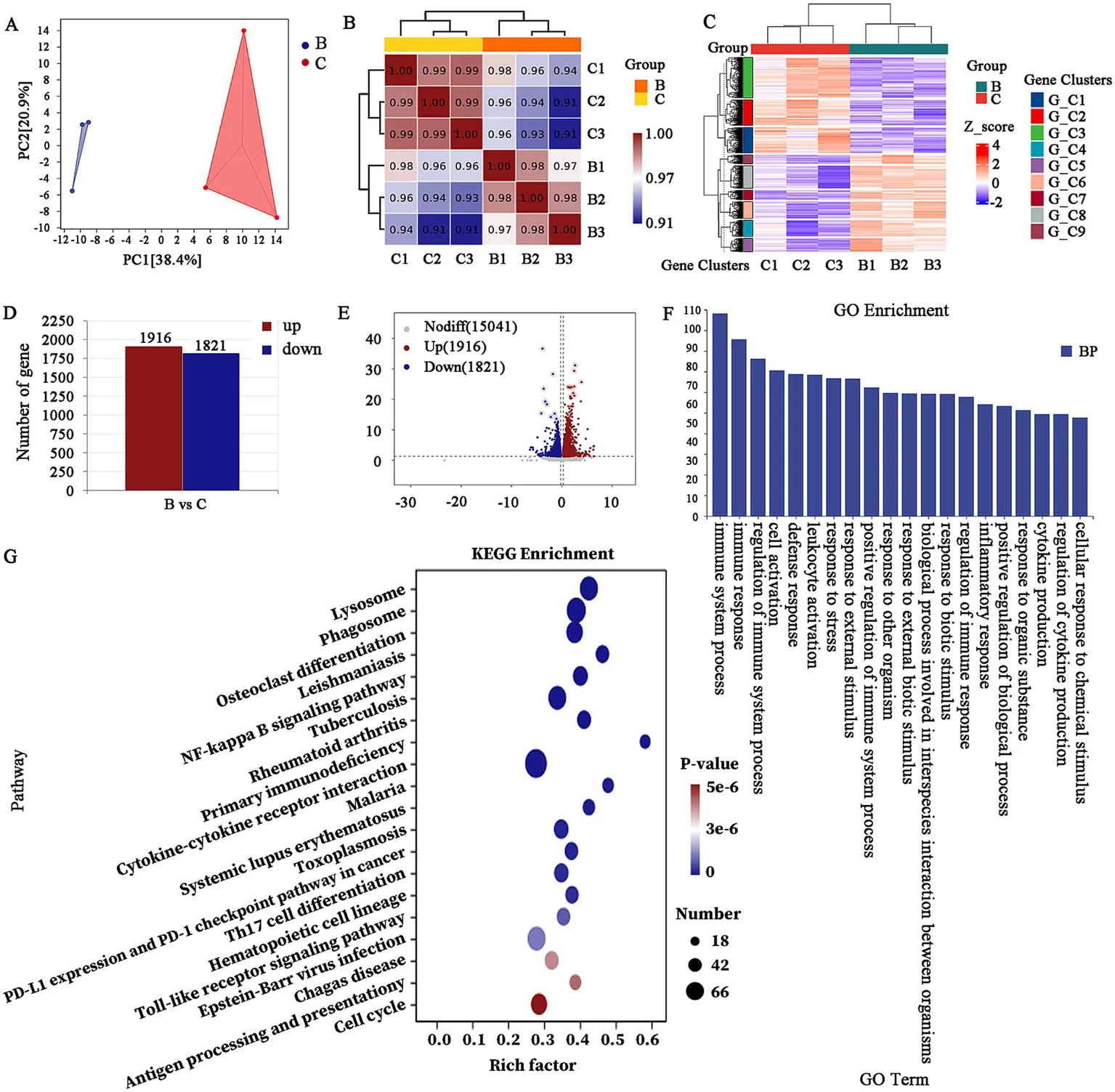
Transcriptome analysis of RNA-Seq data from Ba-medium-dose group vs. PCP untreated group (*n* = 3/group), using the reference genome of *Rattus norvegicus* (http://asia.ensembl.org/Rattus_norvegicus/Info/Index?db=core), including heatmap **(A)**, sample correlation heatmap **(B)**, heatmap **(C)**, statistics of DEGs **(D)**. Vvolcano plot **(E)**, bubble chart of GO enrichment analyses **(F)** and bar plot of KEGG enrichment analyses **(G)**. B, Ba-middle-dose group; C, untreated PCP group; Ba, Baicalin; PCP, *Pneumocystis* pneumonia; PCA, principal component analysis; DEGs, differentially expressed genes; GO, gene ontology; KEGG, kyoto encyclopedia of genes and genomes.

To further characterize the transcriptomic distinctions, PCA and sample correlation analysis were performed, comparing the control group versus the untreated PCP group (Figures 3A,B) and the Ba (middle-dose) group versus the untreated PCP group (Figures 4A,B), respectively. Heatmaps displaying bidirectional cluster analysis revealed the expression patterns of DEGs among groups. Specifically, the heatmaps showed distinct clusters of DEGs between the control and untreated PCP groups (Figure 3C) and between the Ba-middle-dose and untreated PCP groups (Figure 4C). Upregulated genes are represented in red, while downregulated genes are shown in blue. Volcano plots illustrated the following DEG distribution patterns: Between the control group and the PCP untreated group, 5,855 DEGs were identified, including 3,059 upregulated and 2,796 downregulated genes, while the expression of 13,033 genes remained unchanged (Figures 3D,E). Standard gene symbols were successfully annotated for 2,897 out of the 3,059 upregulated DEGs, whereas the remaining 162 genes could not be identified. Between the Ba-middle-dose group and the PCP untreated group, 3,737 DEGs were identified, including 1961 upregulated and 1821 downregulated genes, while 15,041 genes showed no change in expression levels (Figures 4D,E). The RNA-Seq data for the control and Ba-treated groups can be found in Supplementary material 2.

GO enrichment analysis was performed to delineate the functional alterations in response to PCP infection and Ba intervention. The up-regulated DEGs in the PCP model group were significantly enriched in biological processes including “immune system process,” “immune response,” “inflammatory response,” “defense response,” and “response to oxidative stress” (Figure 3F). In contrast, the DEGs regulated by Ba treatment were significantly enriched in distinct biological processes, such as “cytokine production,” “regulation of cytokine production” and “positive regulation of immune response” (Figure 4F).

KEGG pathway enrichment analysis was performed on upregulated DEGs. Compared with the control group, the upregulated DEGs in the untreated PCP group were significantly enriched in pathways including Toll-like receptor signaling, NOD-like receptor signaling, NF-κB signaling, cytokine-cytokine receptor interaction, phagosome, and lysosome. Pathways related to tuberculosis and rheumatoid arthritis were also enriched. Additionally, enrichment was observed for ferroptosis and endoplasmic reticulum stress pathways (Figure 3G). In the Ba-treated group compared to the untreated PCP group, the upregulated DEGs were significantly enriched in pathways including Th17 cell differentiation, antigen processing and presentation, and PD-1/PD-L1 checkpoint signaling. Pathways such as phagosome and NF-κB signaling remained enriched, while ferroptosis was not among the top enriched terms (Figure 4G).

**Figure 5:**
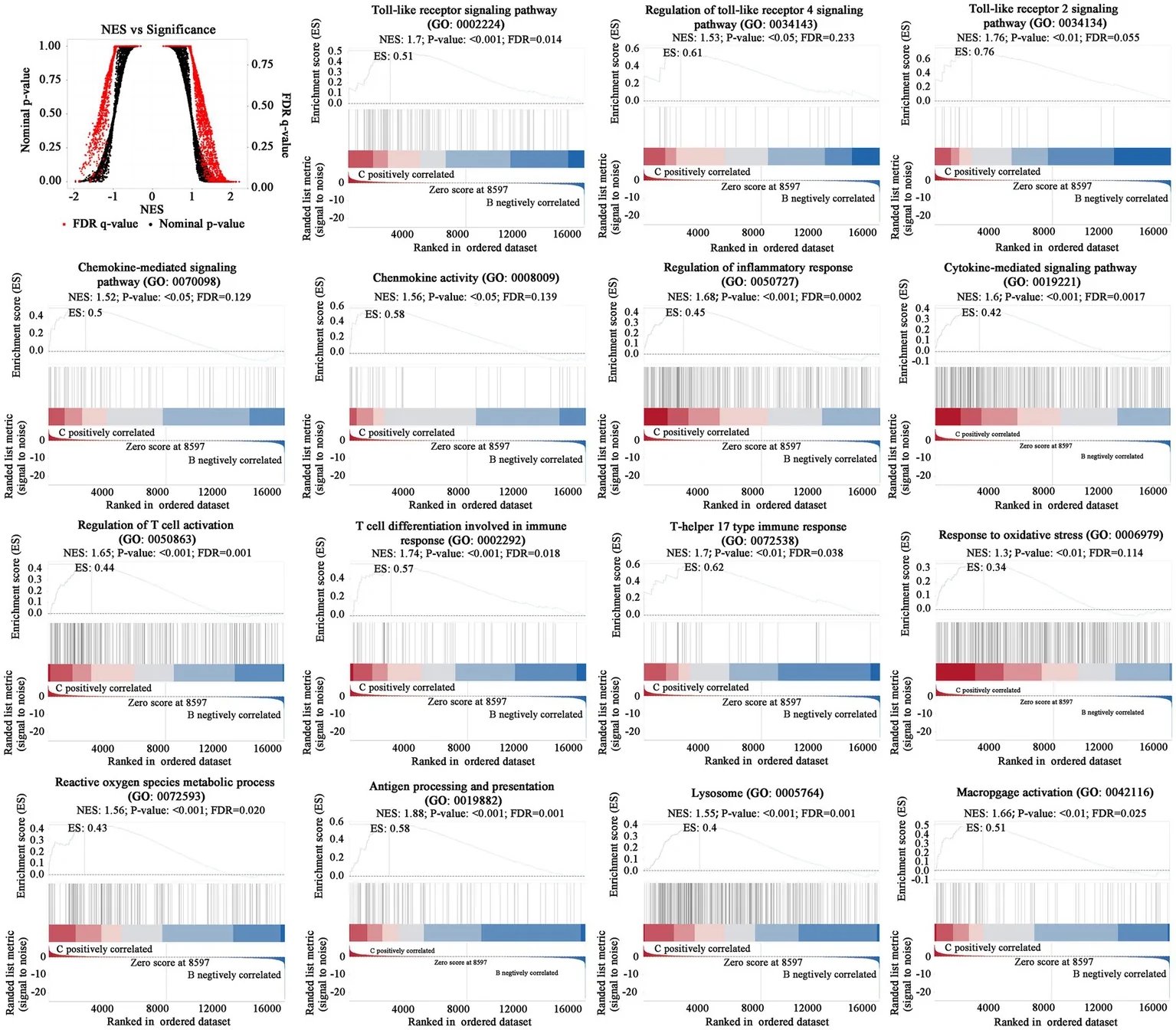
GSEA of GO terms reveals global functional alterations between PCP and Ba medium-dose intervention groups (*n* = 3/group). This figure includes NES and EA plot and summary of significantly enriched GO terms from GSEA. NES plot: A plot comparing the *p*-value with the NES. The horizontal axis represents the NES value, with black dots corresponding to the *p*-value results and red dots corresponding to the FDR results. ES plot: A statistical plot of the ES for gene sets. The horizontal axis represents the ES value, and the vertical axis represents the number of gene sets. The resulting plot displays 16 key signaling pathways that were prominently enriched in the comparison between the untreated PCP group and Ba-medium intervention group, such as GO:0002224, GO:0034143, GO:0034134, GO:0070098, GO:0008009, GO:0050727, GO:0019221, GO:0050863, GO:0002292, GO:0072538, GO:0006979, GO:0072593, GO:0019882, GO:0048002, GO:0005764, and GO:0042116. Schematic representation of the GSEA algorithm: Top panel: Distribution curve of the running ES along the ranked gene list. The peak of the curve (the point farthest from the vertical zero line) represents the final ES for the gene set. Middle panel: Positions of genes belonging to the analyzed gene set within the ranked list. Each vertical tick mark indicates the location of a member gene. Bottom panel: Ranked list metric scores for all genes. Genes are ordered based on their differential expression between the two conditions. Red segments represent genes with higher expression in the treatment group, while blue segments represent genes with higher expression in the control group. GSEA, Gene Set Enrichment Analysis; NES, Normalized Enrichment Score; ES, Enrichment Score; Ba, Baicalin; PCP, *Pneumocystis* pneumonia; GO, Gene Ontology.

To elucidate the molecular mechanisms underlying the therapeutic effect of Ba on PCP, GSEA was performed on the down-regulated DEGs identified from the comparison of the Ba-treated group versus the untreated PCP model group. GO-GSEA results indicated that the significantly down-regulated gene sets were enriched in biological processes related to immune-inflammatory signaling, adaptive immunity, and cellular stress response. Specifically, enriched terms included Toll-like receptor signaling pathway, positive regulation of Toll-like receptor signaling pathway, cytokine- and chemokine-mediated signaling, positive regulation of inflammatory response, T cell activation T cell differentiation, Th17 cell differentiation, antigen processing and presentation, macrophage activation, and response to oxidative stress (Figure 5).

**Figure 6:**
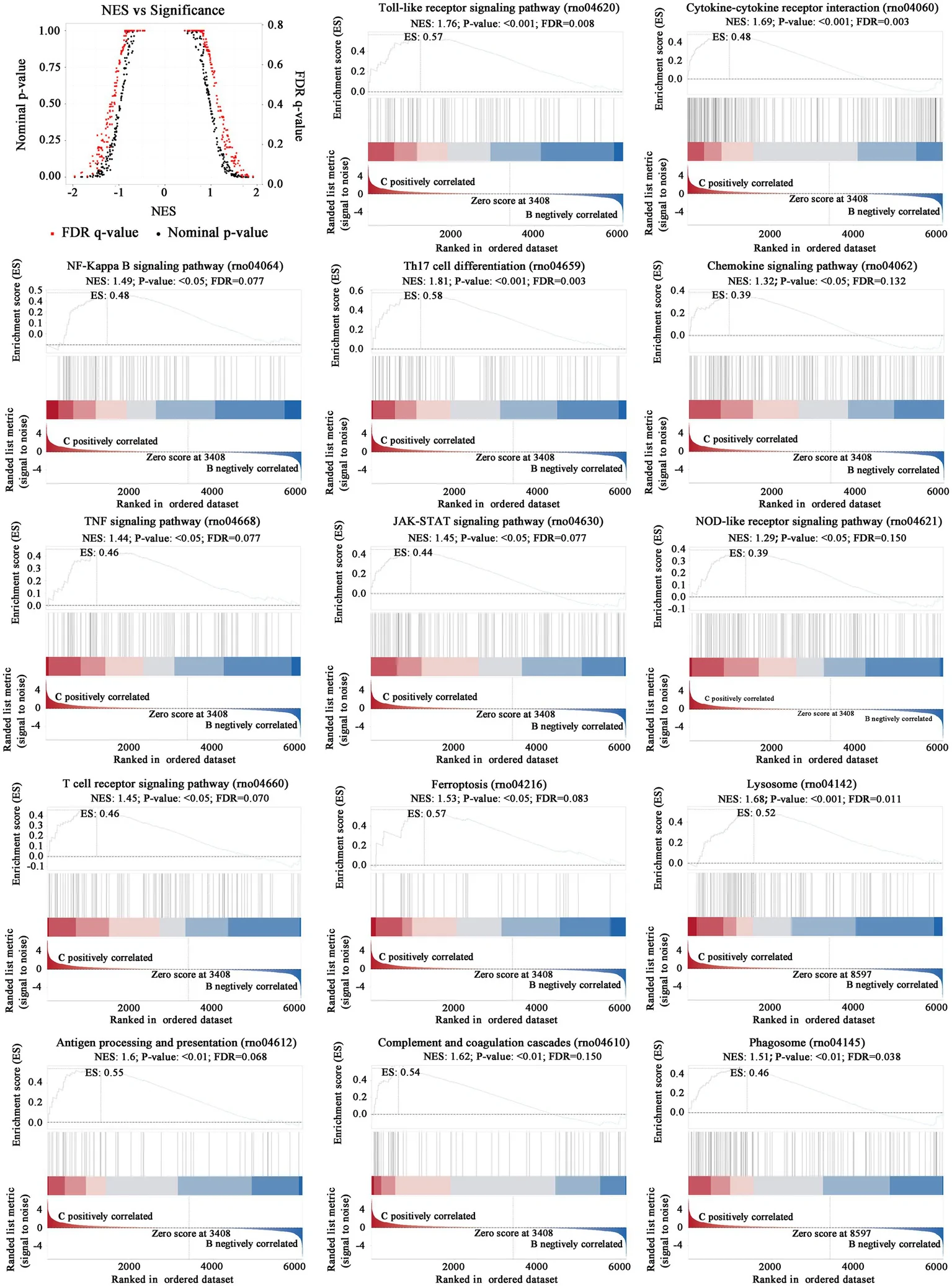
GSEA of KEGG terms reveals global functional alterations between PCP and Ba medium-dose intervention groups (*n* = 3/group). This figure includes NES and EA plot and summary of significantly enriched KEGG terms from GSEA. The NES plot, ES plot, and schematic representation of the GSEA algorithm in this figure follow the same format and interpretation as those presented in Figure 5. The GSEA plot illustrates 14 key KEGG pathways that were prominently enriched in the comparison between the untreated PCP group and Ba medium-dose intervention group, including rno04620, rno04060, rno04064, rno04659, rno04062, rno04668, rno04630, rno04621, rno04660, rno04216, rno04142, rno04612, rno04610, rno04145. GSEA, gene set enrichment analysis; NES, normalized enrichment score; ES, enrichment score; Ba, baicalin; PCP, *Pneumocystis* pneumonia; KEGG, kyoto encyclopedia of genes and genomes.

KEGG-GSEA was performed to complement the GO-GSEA findings. The analysis indicated that Ba intervention was associated with the down-regulation of multiple signaling and immune-related pathways. These included Toll-like receptor signaling, NOD-like receptor signaling, NF-κB signaling, TNF signaling, JAK–STAT signaling, cytokine-cytokine receptor interaction, and chemokine signaling pathways (Figure 6). Down-regulation was also observed for the T cell receptor signaling and Th17 cell differentiation pathways, as well as for pathways related to phagosome, lysosome, complement and coagulation cascades and ferroptosis (Figure 6). The corresponding GSEA tables can be found in Supplementary material 3.

**Figure 7:**
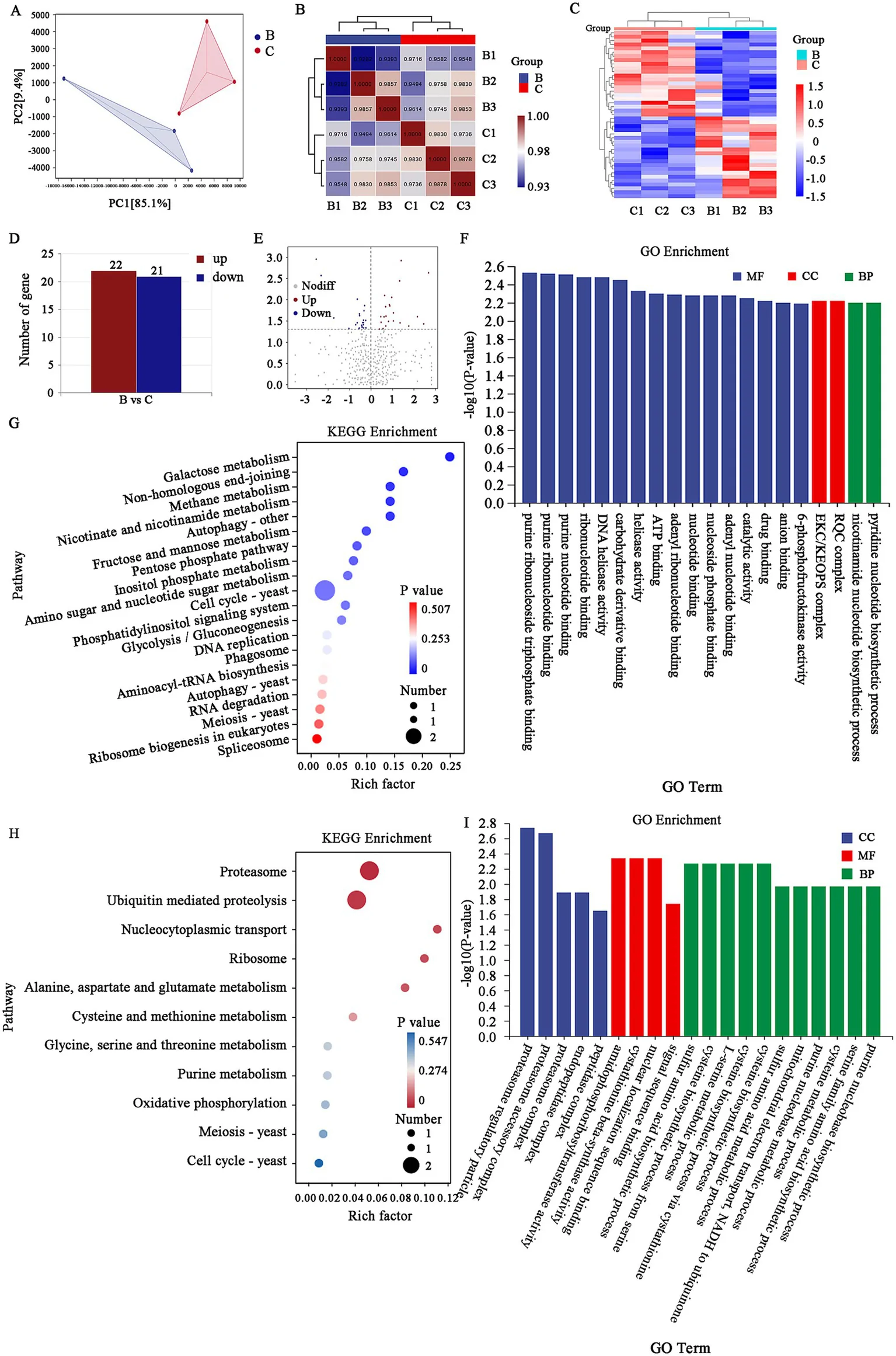
Transcriptome analysis of RNA-seq data from Ba-medium-dose group vs. PCP untreated group (*n* = 3/group), using the reference genome of *Pneumocystis carinii* B80 (https://fungidb.org/fungidb/app/record/dataset/NCBITAXON_1408658), including PCA plot **(A)**, sample correlation heatmap **(B)**, heatmap **(C)**, statistics of DEGs **(D)**. Volcano plot **(E)**, bubble chart of GO enrichment analyses **(F)**, and bar plot of KEGG enrichment analyses of up-regulated DEGs **(G)**. Bubble chart of GO enrichment analyses **(H)** and bar plot of KEGG enrichment analyses of up-regulated DEGs **(I)** of downregulated DEGs. B, Ba-medium-dose group; C, untreated PCP group; Ba, Baicalin; PCP, *Pneumocystis* pneumonia; PCA, principal component analysis; DEGs, differentially expressed genes; GO, gene ontology; KEGG, kyoto encyclopedia of genes and genomes.

To identify potential targets of Ba in *Pneumocystis*, RNA-Seq data from the pathogen were analyzed. DEGs of *Pneumocystis* were identified by comparing the Ba-middle-dose group to the untreated PCP group at the endpoint. PCA and sample correlation analysis are presented in Figures 7A,B, respectively. A heatmap depicting bidirectional clustering of the DEGs is shown in Figure 7C, with upregulated and downregulated genes represented in red and blue, respectively. A total of 22 upregulated and 21 downregulated DEGs were identified (Figure 7D). The volcano plot in Figure 7E illustrates the expression profiles of all genes, categorizing them as upregulated (red), downregulated (blue), or non-differentially expressed (gray). The lists of downregulated and upregulated DEGs are provided in Tables 1, 2, respectively.

**Table 1:** Genes significantly downregulated in the Ba-treated rats.

ID	Gene product description	Expression (standardized FPKM)		Log2foldchange	*p*-value	Swiss-Prot information					
		*P. carinii* extracted from untreated PCP rats	*P. carinii* extracted from PCP rats treated with Ba			The corresponding protein ID in Uniprot database	Organism information	OX=Organism taxonomy	GN=Gene name	PE=Protein Existence	SV=Sequence version
T552_01473	Pre-mRNA-splicing factor ATP-dependent RNA helicase PRP43	41.675	15.657	1.412	1.21E-03	O42945	*Schizosaccharomyces pombe*	284,812	prp43	3	1
T552_00943	tRNA-intron endonuclease	19.380	3.012	2.686	2.35E-03	Q8TFH7	*Schizosaccharomyces pombe*	284,812	sen2	3	1
T552_02144	Asparagine--tRNA ligase	26.040	10.271	1.342	3.64E-03	Q8TFH7	*Schizosaccharomyces pombe*	284,812	slm5	3	1
T552_02964	Extracellular membrane protein CFEM domain-containing protein	396.849	254.630	0.640	8.01E-03	–					
T552_03715	Major surface glycoprotein 2\u00B0C-terminal domain-containing protein	341.327	174.072	0.971	8.87E-03	–					
T552_02494	GTPase grn1	41.060	22.601	0.861	1.32E-02	O74791	*Schizosaccharomyces pombe*	284,812	grn1	1	1
T552_00293	Major surface glycoprotein 2\u00B0C-terminal domain-containing protein	276.403	149.773	0.884	1.41E-02						
T552_02178	Phosphatidylinositol 3-kinase vps34	108.464	67.284	0.689	1.47E-02	P50520	*Schizosaccharomyces pombe*	284,812	vps34	2	2
T552_00219	DASH complex subunit DAM1	28.455	13.741	1.050	2.06E-02	Q6C2T8	*Yarrowia lipolytica*	284,591	DAM1	3	1
T552_00233	tRNA N6-adenosine threonylcarbamoyltransferase	52.936	32.491	0.704	2.46E-02	Q4WDE9	*Neosartorya fumigata*	330,879	kae1	3	1
T552_02028	Chromatin remodeling factor mit1	88.453	64.188	0.463	2.53E-02	Q9P793	*Schizosaccharomyces pombe*	284,812	mit1	1	1
T552_03701	Major surface glycoprotein 2\u00B0C-terminal domain-containing protein	51.068	11.374	2.167	2.53E-02	–					
T552_03287	UDP-N-acetylglucosamine pyrophosphorylase	69.729	45.608	0.612	2.54E-02	O74933	*Candida albicans*	5,476	UAP1	1	1
T552_01550	E3 ubiquitin-protein ligase listerin	57.514	27.787	1.049	3.17E-02	O74349	*Schizosaccharomyces pombe*	284,812	rkr1	3	1
T552_02258	Nicotinate-nucleotide pyrophosphorylase [carboxylating]	67.787	38.431	0.819	3.28E-02	P43619	*Saccharomyces cerevisiae*	559,292	BNA6	1	1
T552_01970	ER lumen protein-retaining receptor 1-A	49.572	35.638	0.476	3.42E-02	Q6PAB8	*Xenopus laevis*	8,355	kdelr1-a	2	1
T552_02179	ATP-dependent DNA helicase II subunit 1	19.624	7.642	1.361	3.53E-02	Q2MHH4	*Aspergillus oryzae*	510,516	ku70	3	1
T552_01389	Major surface glycoprotein 2\u00B0C-terminal domain-containing protein	45.285	8.217	2.462	3.74E-02	–					
T552_01949	DNA endonuclease ctp1	22.464	13.865	0.696	4.19E-02	O74986	*Schizosaccharomyces pombe*	284,812	ctp1	1	2
T552_00171	Ergosterol biosynthetic protein 28	10.554	3.106	1.765	4.21E-02	P40030	*Saccharomyces cerevisiae*	559,292	ERG28	1	1
T552_00514	ATP-dependent 6-phosphofructokinase	130.081	87.904	0.565	4.84E-02	O42938	*Schizosaccharomyces pombe*	284,812	pfk1	1	1
T552_04147	DNA replication licensing factor mcm4	97.131	73.849	0.395	4.96E-02	P29458	*Schizosaccharomyces pombe*	284,812	mcm4	1	2

OX, Organism Taxonomy; GN, Gene name; PE, Protein Existence; SV, Sequence Version; *P. carinii*, *Pneumocystis carinii*; FPKM, Fragments Per Kilobase of transcript per Million mapped reads; ID, identifier.

**Table 2:** Genes significantly upregulated in the Ba-treated rats.

ID	Gene product description	Expression (standardized FPKM)		Log2foldchange	*p*-value	Swiss-Prot information					
		*P. carinii* extracted from untreated PCP rats	*P. carinii* extracted from PCP rats treated with Ba			The corresponding protein ID in Uniprot database	Organism information	OX=Organism Taxonomy	GN=Gene name	PE=Protein existence	SV=Sequence version
T552_03718		169.764	993.493	−2.549	1.12E-03						
T552_02428		3.137	15.679	−2.321	2.72E-03						
T552_01323	26S proteasome regulatory subunit rpn12	53.101	82.431	−0.634	9.80E-03	P50524	*Schizosaccharomyces pombe*	284,812	rpn12	1	1
T552_02053	Amino acid transport system protein	444.616	544.369	−0.292	1.39E-02	P38680	*Neurospora crassa*	OX=367,110	GN=mtr	3	2
T552_01897	E3 ubiquitin-protein ligase ptr1	629.144	786.550	−0.322	1.83E-02	O13834	*Schizosaccharomyces pombe*	OX=284,812	GN=ptr1	1	1
T552_00592		344.554	499.244	−0.535	2.18E-02						
T552_00784	Septation protein 7	65.046	93.803	−0.528	2.39E-02	Q59VX8	*Candida albicans*	OX=237,561	GN=SEP7	1	1
T552_03705		73.295	242.987	−1.729	2.71E-02						
T552_02356	Cystathionine beta-synthase	209.055	265.274	−0.344	3.07E-02	P46794	*Dictyostelium discoideum*	OX=44,689	GN=cysB	2	2
T552_00023		107.696	136.595	−0.343	3.48E-02						
T552_00799	60S ribosomal protein L31	113.119	147.624	−0.384	3.49E-02	Q757D7	*Ashbya gossypii*	OX=284,811	GN=RPL31	3	2
T552_02380	Anaphase-promoting complex subunit 10	50.406	78.372	−0.637	3.51E-02	O42971	*Schizosaccharomyces pombe*	OX=284,812	GN=apc10	1	1
T552_04183	Endocytosis protein end4	295.298	376.854	−0.352	3.90E-02	Q9P6L5	*Schizosaccharomyces pombe*	OX=284,812	GN=end4	1	2
T552_01772	WD repeat-containing protein 62	45.539	74.443	−0.709	3.91E-02	Q3U3T8	*Mus musculus*	OX=10,090	GN=Wdr62	1	2
T552_02416	Amidophosphoribosyltransferase	234.536	315.476	−0.428	4.05E-02	P41390	*Schizosaccharomyces pombe*	OX=284,812	GN=ade4	1	1
T552_01729	Nucleoporin nup45	65.981	87.810	−0.412	4.39E-02	Q09793	*Schizosaccharomyces pombe*	OX=284,812	GN=nup45	1	2
T552_00738	Probable coatomer subunit zeta	61.088	80.831	−0.404	4.53E-02	O74891	*Schizosaccharomyces pombe*	284,812	GN=ret3	3	1
T552_00170	Probable hydrolase nit2	278.679	352.454	−0.339	4.64E-02	O94660	*Schizosaccharomyces pombe*	284,812	nit2	3	1
T552_02097		36.251	52.827	−0.543	4.68E-02						
T552_01488		8.159	16.599	−1.025	4.80E-02						
T552_01403	26S proteasome regulatory subunit 6B homolog	74.396	109.479	−0.557	4.84E-02	O74894	*Schizosaccharomyces pombe*	284,812	rpt3	3	1

OX, Organism Taxonomy; GN, Gene name; PE, Protein Existence; SV, Sequence Version; *s*, *Pneumocystis carinii*; FPKM, Fragments Per Kilobase of transcript per Million mapped reads; ID, identifier.

GO enrichment analysis mapped the upregulated DEGs to specific Molecular Function (MF), Cellular Component (CC), and Biological Process (BP) terms. Among the top enriched biological processes, terms related to the negative regulation of nucleotide metabolic processes and nucleotide biosynthetic pathways were prominent (Figure 7F). KEGG pathway enrichment analysis for the upregulated DEGs identified significant associations, with the “cell cycle—yeast” pathway being the most significantly enriched term (Figure 7G). GO and KEGG pathway enrichment analyses for the downregulated DEGs are summarized in Figures 7H,I, respectively. The downregulated DEGs were significantly enriched in pathways related to glycolysis/gluconeogenesis, pentose phosphate pathway, amino acid metabolism, and nucleotide metabolism.

### 3.2 Identification of potential Ba targets against PCP through an integrative approach

#### 3.2.1 Identification of overlapping drug-disease targets

a. Identification of Ba-related targets: The canonical SMILES structure of Ba (C1=CC=C(C=C1)C2=CC(=O)C3=C(C(=C(C=C3O2)O[C@H]4C@@HO)O)O) was retrieved from the PubChem database. Potential targets of Ba were predicted using the SwissTargetPrediction, STITCH, ChEMBL, and SEA databases, yielding 100, 26, 47, and 71 targets, respectively. The corresponding data are presented in Supplementary material 4A (see sheets: Ba_SwissTargetPrediction, Ba_STITCH, Ba_ChEMBL, and Ba_SEA). All retrieved targets were standardized to official gene symbols using the UniProt database. After integrating the results from all databases and removing duplicates, a total of 115 unique potential targets of Ba were obtained. b. Screening of PCP-related targets: As illustrated in Supplementary material 4B, potential targets associated with PCP were retrieved by searching the GeneCards, OMIM, NCBI, Open Targets Platform, and CTD databases using the keyword “*Pneumocystis* pneumonia,” which yielded 1,415, 374, 18, 875, and 8,769 targets, respectively. Following the integration of these datasets and removal of duplicate entries, 9,694 non-redundant potential targets for PCP were identified. c. Identification of common drug-disease targets: The 115 potential targets of Ba were cross-referenced with the 9,694 potential targets of PCP to identify common targets. A Venn diagram was constructed, revealing 93 overlapping targets (Supplementary materials 4C,D), which were considered as the putative therapeutic targets of Ba against PCP for subsequent investigations.

#### 3.2.2 Validation and refinement of core targets using transcriptomic data

**Figure 8:**
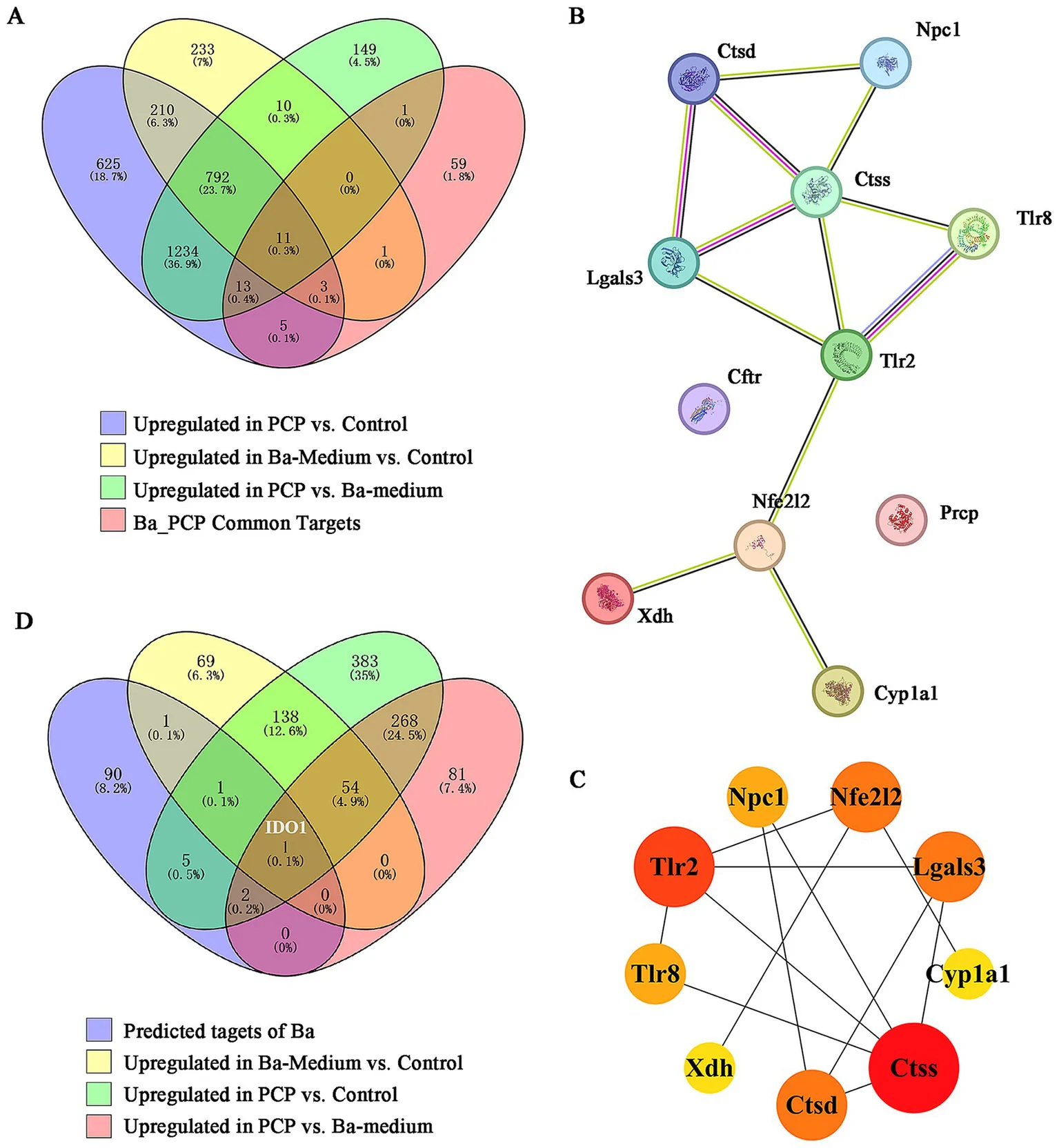
Validation and identification of core therapeutic targets through transcriptomic integration. **(A)** Venn diagram of overlapping targets. Eleven candidate targets were identified at the intersection of four gene sets: drug-disease targets from network pharmacology (*n* = 93), and three sets of differentially expressed genes (|log₂FC| > 1.2, *p* < 0.05) from RNA-Seq comparisons (PCP vs. control, Ba-treated vs. control, PCP vs. Ba-treated). (B, C) PPI network of candidate targets. **(B)** Initial PPI network of the 11 genes constructed using STRING (confidence > 0.4). Two genes (Cftr, Prcp) were disconnected and excluded. **(C)** Final PPI network consisting of nine interconnected core proteins (Xdh, Nfe2l2, Cyp1a1, Tlr8, Tlr2, Ctss, Lgals3, Npc1, Ctsd), representing key mediators of Ba′s anti-PCP effects. **(D)** Venn diagram of candidate targets identified under stringent criteria. The diagram illustrates the intersection of four gene sets: differentially expressed genes (|log₂FC| > 2, *p* < 0.05) from three RNA-Seq comparisons (PCP vs. control, Ba-treated vs. control, PCP vs. Ba-treated) and Ba′s predicted targets from the SwissTargetPrediction database. This intersection highlights high-confidence targets likely involved in Ba′s anti-PCP mechanism. PPI, protein–protein interaction, Ba, baicalin; PCP, *Pneumocystis* pneumonia.

**Figure 9:**
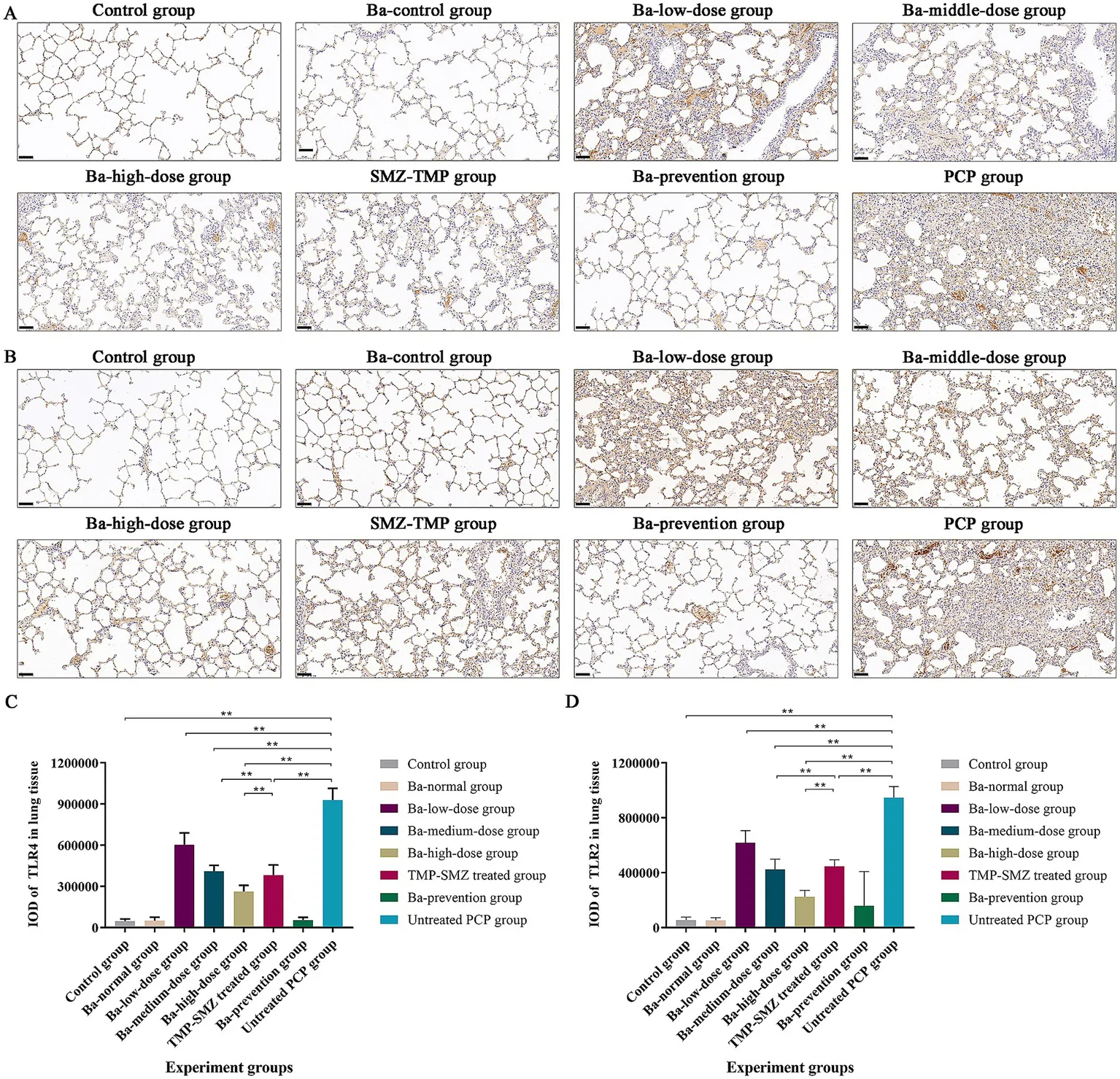
The anti-PCP effect of Ba activates the protein expression of TLR4 and TLR2 in lung tissue. **(A,B)** IHC assay data showed the expression levels and distribution of TLR4/2 protein in pulmonary tissue derived from various groups (magnification, ×200; scale bar = 50 μm) and **(C,D)** corresponding statistical results. Ba, baicalin; PCP, *Pneumocystis* pneumonia; TLR, Toll-like receptor. **p < 0.01, *n*= 7/group.

Venn analysis was performed between the drug-disease target set (93 genes identified in Supplementary materials 4C,D) and DEGs derived from rat RNA-Seq data, which were screened under the criteria of |log₂ fold change| > 1.2 and adjusted *p* < 0.05. This analysis aimed to identify potential key targets through which Ba exerts its anti-PCP effects. As shown in Figures 8A, 9 overlapping genes were identified at the intersection of the following four gene sets: (1) the 93 drug-disease targets from network pharmacology; (2) genes upregulated in the untreated PCP group compared to the control group; (3) genes upregulated in the Ba-treated group compared to the control group; and (4) genes upregulated in the untreated PCP group compared to the Ba-treated group (equivalently, genes downregulated in the untreated PCP group compared to the Ba-treated group). These 11 candidate targets include: Nfe2l2 (Nrf2), Ctss, Ctsd, Tlr8, Tlr2, Cyp1a1, Xdh, Npc1, Cftr, Lgals3, and Prcp. The PPI network analysis of the 11 candidate targets was performed. Following filtering criteria with a confidence score > 0.4 and removal of disconnected nodes, the final network visualized the interactions among nine core proteins: Xdh, Nfe2l2, Cyp1a1, Tlr8, Tlr2, Ctss, Lgals3, Npc1, and Ctsd (Figures 8B,C). Detailed data from the STRING database supporting the PPI network analysis are provided in Supplementary material 5. They represent high-confidence candidates implicated in the therapeutic mechanism of Ba against PCP. Among these nine core genes, Nrf2 occupied a central position within the network, functioning as a pivotal hub molecule that interconnects oxidative stress, inflammatory response, and immunomodulation. In the defense against oxidative damage in lung tissue, Nrf2 orchestrates a comprehensive regulatory role that cannot be substituted by the other eight genes. Based on its hub status in the network and its well-established biological functions, Nrf2 was selected as the core target for subsequent investigation into the mechanism of Ba against PCP.

To identify the most robustly modulated targets, we applied a more stringent differential expression threshold (|log2FoldChange| > 2, *p* < 0.05). Intersection analysis revealed that indoleamine 2,3-dioxygenase 1 (IDO1) was the sole gene common to the predicted targets of Ba (from the SwissTargetPrediction database) and the upregulated DEGs identified in the following comparisons: control vs. untreated PCP group, control vs. Ba-treated group, and Ba-treated vs. untreated PCP group. This analysis, visualized using the Venny online tool, consistently pinpointed IDO1 as the top candidate under the stringent criterion (Figure 8D). Consequently, IDO1 was selected for further experimental validation.

### 3.3 Suppression of IDO1 expression observed following Ba treatment in *Pneumocystis*-infected immunosuppressed rats

**Figure 10:**
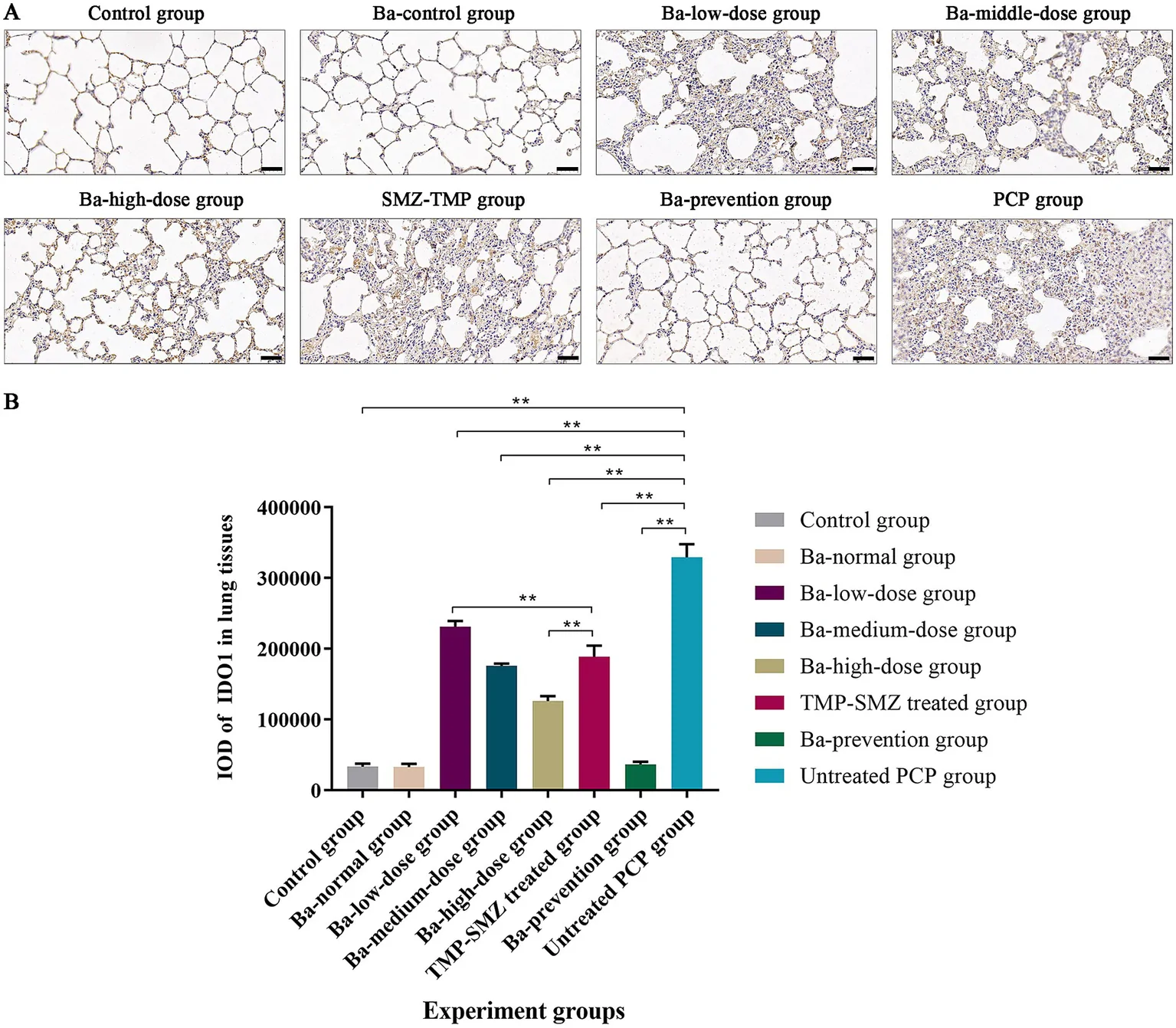
Suppression of IDO1 expression observed following Ba treatment in *Pneumocystis*-infected immunosuppressed rats. **(A)** IHC assay data showed the expression levels and distribution of IDO1 protein in lung tissue derived from various groups (magnification, ×200; scale bar = 50 μm) and **(B)** corresponding statistical results. Ba, baicalin; IDO1, indoleamine 2,3 dioxygenase 1; IOD, integrated optical density. **p <0.01, *n* = 7/group.

Through bioinformatics analysis aforementioned, IDO1 was identified as a potential target of Ba against PCP. To validate these results, the expression levels of IDO1 in lung tissues from different experimental groups (7 samples per group) were assessed using IHC. IDO1 expression was primarily localized to respiratory epithelial cells and a few other cell types in the PCP-untreated group. In contrast, weak or negligible IDO1 staining was observed in the control and Ba-treated groups (Figure 10A). Quantitatively, IDO1 expression levels significantly decreased in the Ba treatment group (*p* < 0.01) and the TMP-SMZ treatment group (*p* < 0.01) compared with the PCP-untreated group. No significant differences in IDO1 expression were noted between the control group and the Ba-normal or Ba-prevention groups (*p* > 0.05). Moreover, among the three Ba-treated groups, IDO1 expression levels appeared to be concentration-dependent. The corresponding integrated optical density (IOD) values for IDO1 exhibited consistent trends, further supporting these observations (Figure 10B).

### 3.4 Ba exerts therapeutic and preventive effects in *Pneumocystis*-infected immunosuppressed rats via potential activation of Nrf2 Signaling

**Figure 11:**
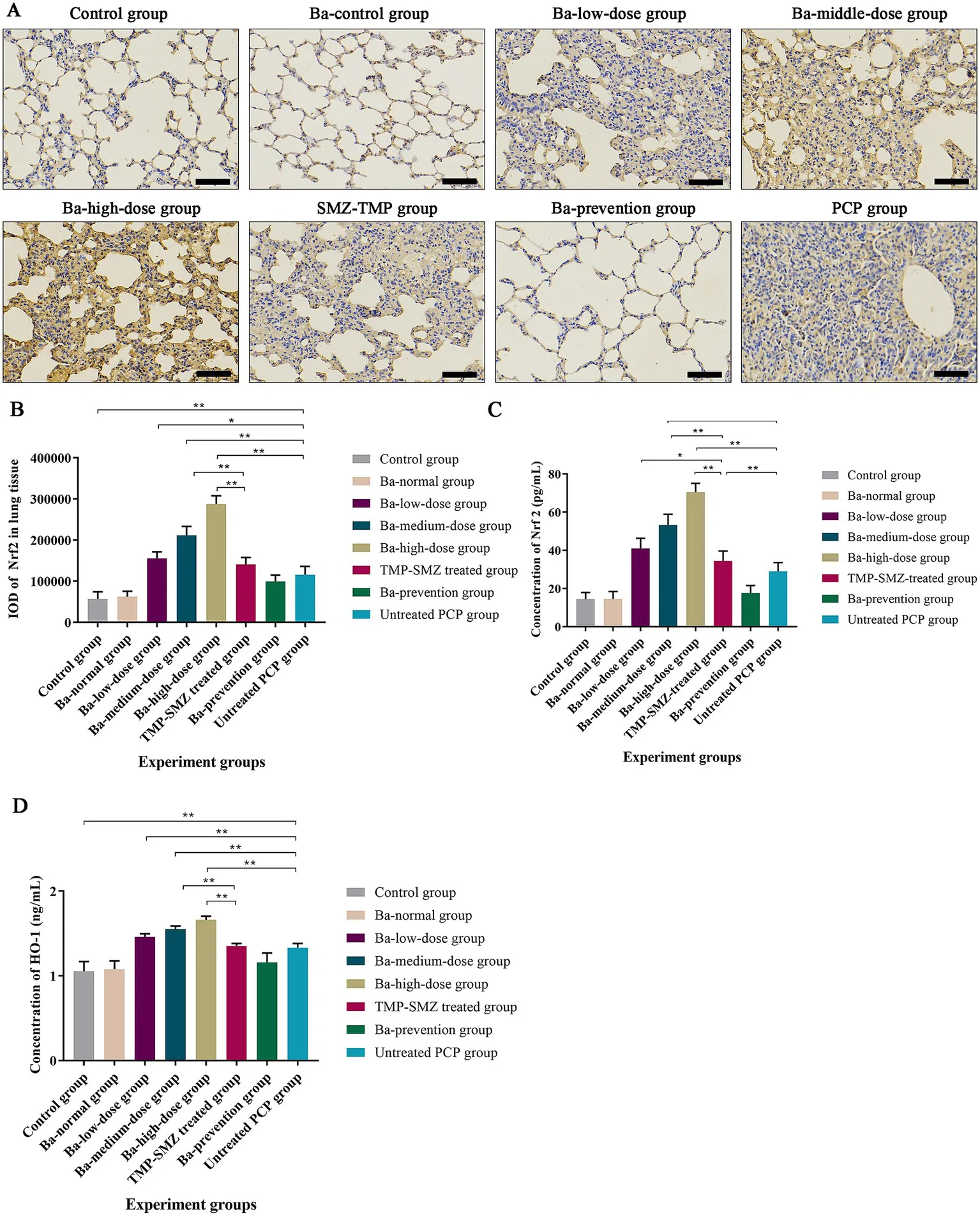
The anti-PCP effect of Ba activates the protein expression of Nrf2 in lung tissue **(A)** IHC assay data showed the expression levels and distribution of Nrf2 protein in pulmonary tissue derived from various groups (magnification, ×200; scale bar = 50 μm) and **(B)** corresponding statistical results. **(C)** Nrf2 protein expression levels detected by ELISA in experimental groups. **(D)** HO-1 protein expression levels detected by ELISA in experimental groups. Nrf2, nuclear factor erythroid 2-related factor 2; IOD, integrated optical density; HO-1, heme oxygenase-1; Ba, Baicalin; PCP, *Pneumocystis* pneumonia. *p < 0.05, **p < 0.01, *n* = 7/group.

The expression of the Nrf2 protein was evaluated using IHC (7 samples per group). In the PCP untreated group, the Nrf2 protein was predominantly localized in bronchial epithelial cells and certain types of inflammatory cells (Figure 11A). Importantly, the integrated optical density (IOD) value of Nrf2 was significantly higher in both the Ba treatment group and the Ba prevention group compared with the PCP untreated group (Figure 11B). The Nrf2 and HO-1 concentrations detected by ELISA in each sample were consistent with the IHC findings (Figures 11C,D).

### 3.5 Ba modulates the toll-like receptor signaling pathway by down-regulating TLR2 and TLR4 expression in the lung

Based on both GO and KEGG enrichment analyses, and GO-GSEA and KEGG-GSEA analyses, the TLR signaling pathway was identified as a primary pathway potentially modulated by Ba in the treatment of PCP. TLR2 and TLR4 serve as critical upstream sensors within this pathway, we next examined their protein expression levels in the lung tissues of each experimental group using IHC (7 samples per group). In lung sections from the PCP-untreated control group, TLR4 and TLR2 expression was predominantly observed in alveolar macrophages and a limited number of other cell types. In contrast, significantly weaker or nearly absent immunostaining for both TLR4 and TLR2 was detected in the PCP model group and the Ba-treated groups (Figures 9A,B). Quantitative analysis demonstrated that the expression levels of TLR4 and TLR2 were significantly reduced in the Ba-treated group and the TMP-SMZ-treat-group compared with the untreated-PCP group (*p* < 0.01 for both). However, no significant differences in TLR4 or TLR2 expression were observed between the control group and either the Ba normal-dose group or the Ba prevention group (*p* > 0.05). Furthermore, across the three Ba-treated groups, the expression of TLR4 and TLR2 exhibited a concentration-dependent pattern. The corresponding integrated optical density (IOD) values for TLR4 and TLR2 showed a consistent trend, further corroborating these findings (Figures 9C,D).

### 3.6 Ba inhibits the expression of the *Pneumocystis Rtt109* gene in pulmonary tissues of *Pneumocystis*-infected immunosuppressed rats

Based on bioinformatics analyses of RNA-Seq data reflecting physiological changes in *Pneumocystis* following Ba treatment, combined with findings from existing literature, *PcRtt109* was identified as a potential therapeutic target for PCP (Dahlin et al., 2014; Sugumar et al., 2016). To validate this, the expression of the *PcRtt109* gene in pulmonary tissues of *Pneumocystis*-infected immunosuppressed rats was assessed (7 samples per group).

**Figure 12:**
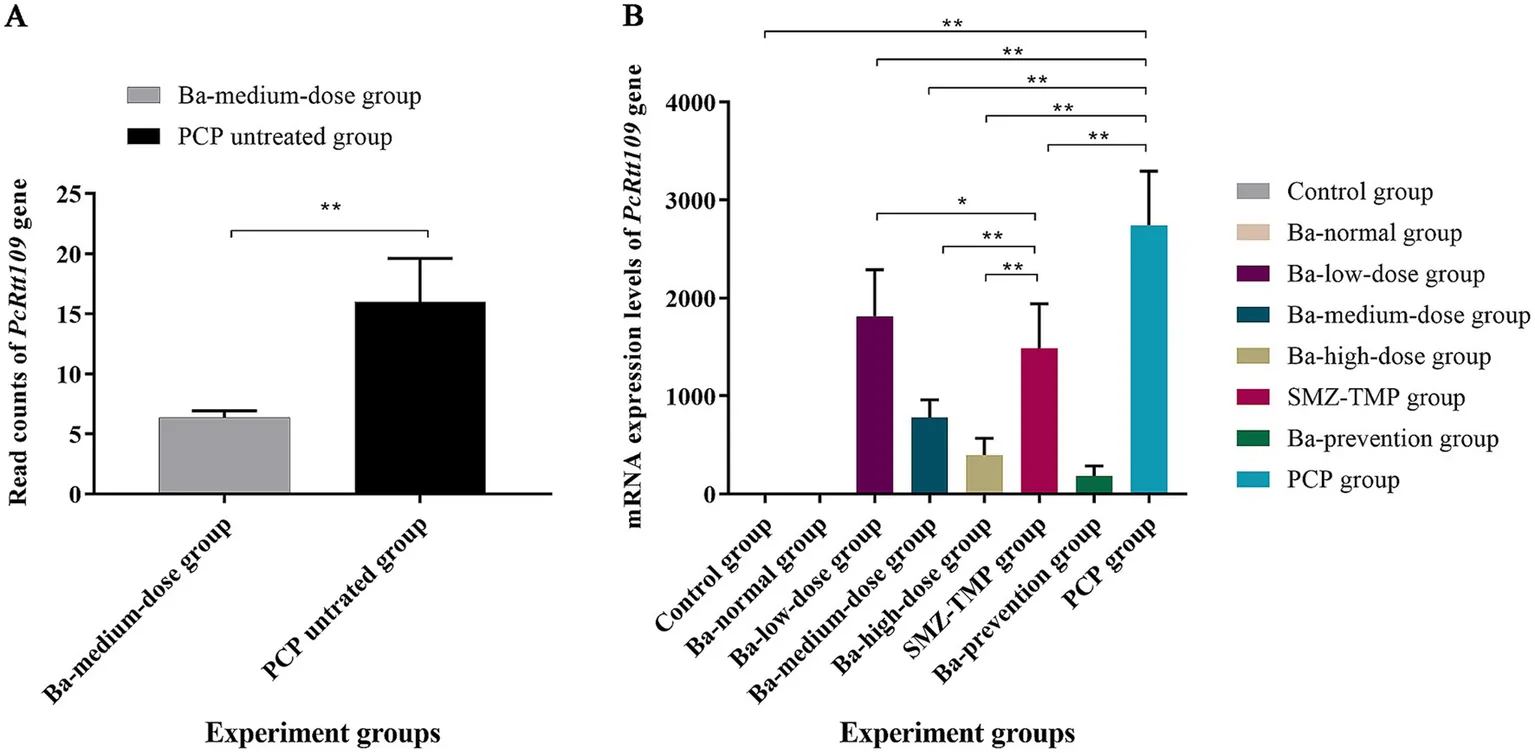
The expression levels of *PcRtt109* in lung tissues. **(A)** The read counts of *PcRtt109* gene in Ba-middle-dose group and PCP untreated group from RNA-Seq data. **(B)** The expression levels of *PcRtt109* gene lung tissues from different experimental groups. *PcRtt109, Pneumocystis carinii* Rtt*109*. *p < 0.05, **p < 0.01, *n* = 7/group.

RNA-Seq read counts revealed that *PcRtt109* expression was markedly downregulated following Ba treatment (Figure 12A). Quantitative real-time PCR (RT-qPCR) further indicated these findings, showing that *PcRtt109* mRNA expression levels in the lung tissues of the PCP untreated group were significantly upregulated compared to the Ba-treated group. Moreover, the suppression of *PcRtt109* expression in the Ba-treated group was found to be dose-dependent (Figure 12B).

### 3.7 Network features of cytokines affected by Ba in its anti-PCP mechanism

**Figure 13:**
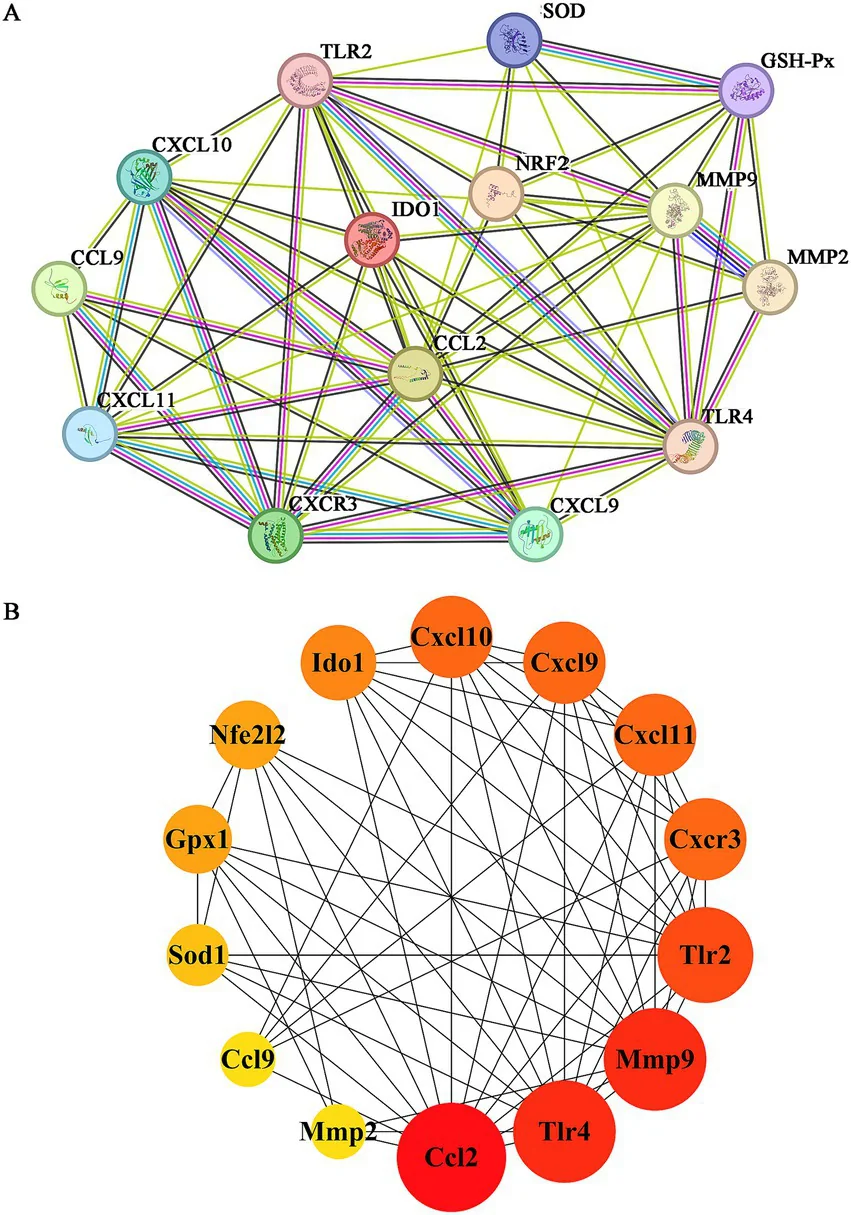
PPI interaction network analysis of the 14 DEGs detected in our studies including IDO1, Nrf2, chemokines (CCL2, CCL9, CXCR3, CXCL9, CXCL10, CXCL11)—oxidative stress biomarkers (SOD, GSH-Px), key members in the TLR signaling pathway (TLR2 and TLR4) and matrix metalloproteinases (MMPs, MMP2 and MMP9) using the String online database **(A)** and corresponding final PPI network **(B)**. IDO1, indoleamine 2,3 dioxygenase 1; Nrf2, nuclear factor erythroid 2-related factor 2; CCL2, chemokine (C-C motif) ligand 2 (also known as monocytic chemotactic protein 1, MCP-1); CCL9, chemokine (C-C motif) ligand 9; CXCR3, C-X-C motif receptor 3; CXCL9, C-X-C motif ligand 9; CXCL10, C-X-C motif ligand 10; CXCL11, C-X-C motif ligand 11; SOD, superoxide dismutase; GSH-Px, glutathione peroxidase; TLR, toll-like receptor; matrix metalloproteinase-2; MMP9, matrix metalloproteinase-9.

A PPI network analysis was performed to investigate the interactions among 14 key proteins implicated in our study, including IDO1, Nrf2, chemokines (CCL2, CCL9, CXCR3, CXCL9, CXCL10, and CXCL11), oxidative stress biomarkers (SOD and GSH-Px), key members in the TLR signaling pathway (TLR2 and TLR4) and matrix metalloproteinases (MMP2 and MMP9). The PPI network was constructed using the STRING online database (Figure 13A). A PPI network was subsequently constructed to visualize the interactions among these 14 potential targets, applying a filtering criterion of a confidence score > 0.4 (Figure 13B). The detailed data from the STRING database supporting this PPI network analysis are provided in Supplementary material 6. The observed interactions further support the multi-target mechanism underlying the anti-PCP effects of Ba.

### 3.8 Molecular docking predicts favorable binding potential of Ba to Nrf2, IDO1, TLR2, TLR4, and *PcRtt109*

**Figure 14:**
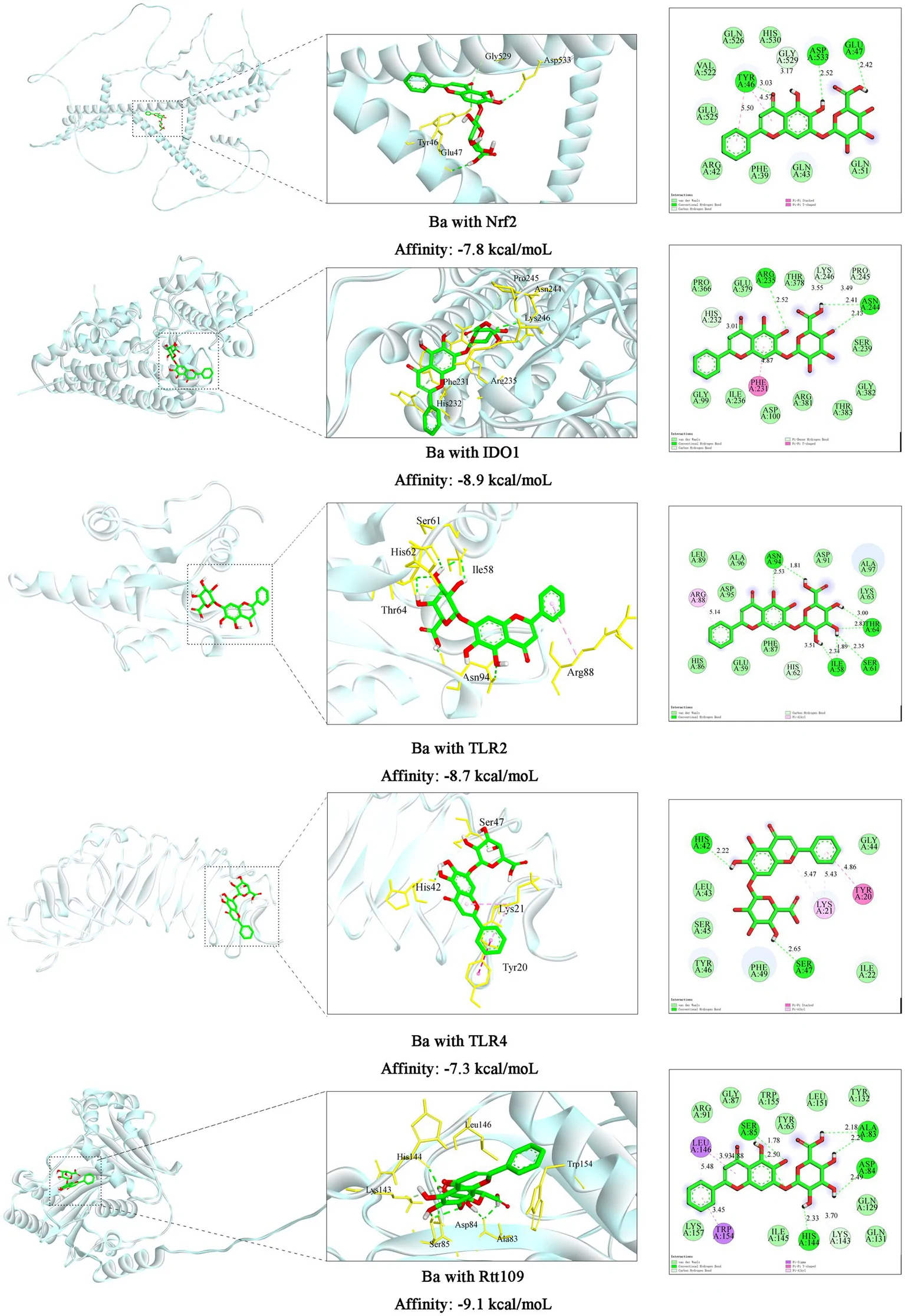
Molecular docking results of Ba into the protein crystal structures of Nrf2, IDO1, TLR2, TLR4, and *PcRtt109*. IDO1, Indoleamine 2,3 Dioxygenase 1; Nrf2, Nuclear Factor Erythroid 2-Related Factor 2; *PcRtt109, Pneumocystis carinii* Rtt*109*; IDO1, indoleamine 2,3 dioxygenase 1; TLR, toll-like receptor; Ba, baicalin.

Molecular docking, a well-established *in silico* structure-based technique, is widely applied in drug discovery to predict binding interfaces between target proteins (enzymes) and drug molecules (ligands) (Pinzi and Rastelli, 2019; Saikia and Bordoloi, 2019; Stanzione et al., 2021). In this study, AutoDock Vina was utilized to perform molecular docking of Ba with the crystal structures of Nrf2, IDO1, TLR2, TLR4, and *PcRtt109*. The docking results revealed that the binding affinities of compound Ba with Nrf2, IDO1, TLR2, TLR4, and *PcRtt109* (−7.8, −8.9, −8.7, −7.3, and −9.1 kcal/mol, respectively) all exceeded the threshold of −7.0 kcal/mol, which is commonly considered indicative of strong binding. These findings suggested potential interactions between Ba and the candidate target proteins, with *PcRtt109* showing the most favorable docking score (Figure 14). The protein sources, molecular docking parameters, and the corresponding docking binding sites are provided in Supplementary material 7, providing a critical structural context for the interpreted binding affinities. These computational predictions suggest that Ba could interact with these potential target proteins, which is consistent with the hypothesis that its therapeutic effects against PCP might involve the modulation of these targets.

### 3.9 Stability and molecular interactions of Ba with IDO1, Nrf2, TLR2/4, and *PcRtt109* characterized by molecular dynamics simulations

**Figure 15:**
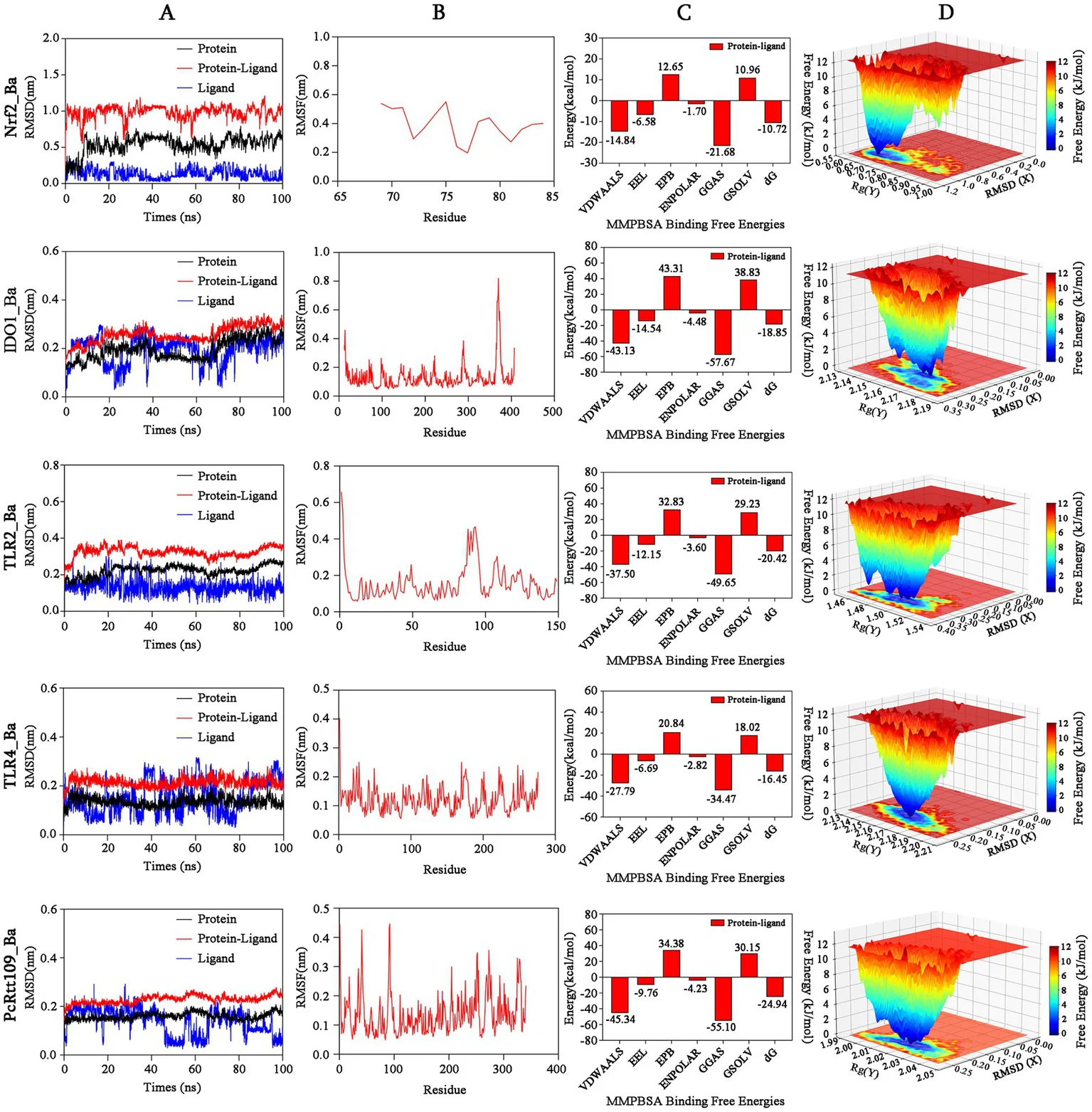
Molecular dynamics simulations of the binding mode and stability of Ba in potential target protein complexes. **(A)** Conformational stability profiling via RMSD analysis. **(B)** Local flexibility profiling via RMSF analysis. **(C)** Energetic contribution decomposition via per-residue free energy analysis. **(D)** 3D free energy surface construction via Gibbs landscape analysis. RMSD, root-mean-square deviation; RMSF, root-mean-square fluctuation; 3D, three-dimensional; IDO1, indoleamine 2,3 dioxygenase 1; Nrf2, nuclear factor erythroid 2-related factor 2; *PcRtt109*, *Pneumocystis carinii* Rtt109; IDO1, indoleamine 2,3 dioxygenase 1; TLR, toll-like receptor; Ba, baicalin.

The stability and conformational dynamics of the simulated complexes were rigorously evaluated by analyzing the trajectories. Key metrics, including the root mean square deviation (RMSD) of the protein backbone and the ligand, root mean square fluctuation (RMSF) of residue flexibility, radius of gyration (Rg), and solvent-accessible surface area (SASA), were calculated. Furthermore, protein-ligand interactions were quantified by monitoring intermolecular hydrogen bonds, and the binding affinity was assessed using the Molecular Mechanics/Poisson-Boltzmann Surface Area (MMPBSA) method. The simulations revealed that the RMSD values for all five complexes converged within 10–40 ns, demonstrating rapid system stabilization and stable trajectories (Figure 15A). This dynamic stability was paralleled by structural rigidity, evidenced by low RMSF values (Figure 15B), increased molecular compactness from reduced Rg (Supplementary material 8A) and SASA (Supplementary material 8B), and persistent intermolecular hydrogen bonds (Supplementary material 8C). Energetically, the MM/PBSA-derived ΔGbind values demonstrated highly favorable interactions between Ba and the potential targets: −10.72 kcal/mol (Nrf2), −18.85 kcal/mol (IDO1), −20.42 kcal/mol (TLR2), −16.45 kcal/mol (TLR4), and −24.94 kcal/mol (*PcRtt109*), with the latter exhibiting the strongest binding affinity (Figure 15C). The existence of a dominant low-energy basin in the free energy landscape for each complex provides conclusive evidence of thermodynamic stability (Figure 15D and Supplementary material 8D). Therefore, the computational data suggest that Ba has the capability for stable, high-affinity binding to a diverse range of the investigated protein targets.

## 4 Discussion

*Pneumocystis*, an opportunistic fungal pathogen, is a major cause of PCP in immunocompromised individuals, including patients with or without HIV infection (Ma et al., 2018; Xue et al., 2023; McDonald et al., 2024). The prevalence of PCP has shown an increasing trend in recent years, largely due to the growing use of immunosuppressive therapies (Weyant et al., 2021; Xue et al., 2023). While trimethoprim–sulfamethoxazole (TMP–SMX) remains the first-line treatment and prophylactic agent for PCP, its clinical utility is limited by the emergence of dihydropteroate synthase (dhps) mutant strains conferring drug resistance, as well as by intolerance and allergic reactions (Ma et al., 2018; Weyant et al., 2021; Haseeb et al., 2022). Thus, there is an urgent need to develop novel therapeutics with enhanced efficacy and reduced side effects for PCP management.

In this study, we investigated the molecular mechanisms underlying the therapeutic and prophylactic effects of Ba against PCP using dual RNA-Seq technology. Our findings demonstrate that Ba significantly downregulates the expression of IDO1 and *PcRtt109* in *Pneumocystis*-infected immunosuppressed rats, suggesting IDO1 as a key host target and providing additional support for the role of *PcRtt109* as a candidate of Ba (Kottom et al., 2011). In addition, Ba treatment was associated with increased Nrf2 protein expression, which is consistent with potential activation of the Nrf2 pathway. These results support the therapeutic potential of Ba in targeting multiple pathways and components of the host-pathogen interface.

### 4.1 Molecular mechanisms of Ba against PCP

Recent advances in RNA sequencing (RNA-Seq) have revolutionized transcriptomic studies, enabling rapid and comprehensive exploration of differential gene expression (DEG) profiles, disease biomarkers, and therapeutic targets (Oshlack et al., 2010; Stark et al., 2019; Hong et al., 2020; Smail and Montgomery, 2024). Dual RNA-Seq, which simultaneously reveals both host and pathogen transcriptomes, provides critical insights into host-pathogen interactions, underlying physiological changes, and hidden pathogenic mechanisms undetectable by traditional assays (Westermann et al., 2012; Westermann et al., 2017; Westermann and Vogel, 2021). Based on this approach, our study revealed several key molecular pathways influenced by Ba.

Integrated analysis of GO and KEGG pathway enrichment from host transcriptomic data delineates a multi-layered understanding of the immunopathogenesis of PCP and the restorative action of Ba. In the untreated PCP model, both GO and KEGG enrichment convergently suggest a state of innate immune hyperactivation and inflammatory dysregulation, marked by upregulation of processes such as “inflammatory response” and pathways including NF-κB signaling and cytokine-cytokine receptor interaction. The concurrent enrichment of ferroptosis and endoplasmic reticulum stress pathways further reflects significant oxidative and cellular damage. The therapeutic intervention with Ba likely induced a selective reprogramming of this pathological landscape. KEGG analysis revealed a critical downregulation of the cytokine-cytokine receptor interaction pathway, which aligns with our experimental observation of reduced levels of key pro-inflammatory chemokines (e.g., CCL2, CXCL9, CXCL10) (Xue et al., 2025). This attenuation of the cytokine storm was accompanied by a shift in enrichment towards pathways involved in adaptive immune regulation, such as Th17 cell differentiation, antigen processing and presentation, and PD-1/PD-L1 checkpoint signaling, suggesting a modulatory effect on T-cell responses and potential attenuation of hyperinflammation. Notably, while certain innate immune and inflammatory pathways (e.g., NF-κB signaling) remained enriched, implying preserved antimicrobial defense. The absence of ferroptosis among the top enriched terms suggests that Ba may alleviate oxidative stress-associated cell injury. These findings imply that Ba does not broadly suppress immunity but rather reprograms the host response: it simultaneously dampens the maladaptive, innate immunity-driven hyperinflammation and associated oxidative stress, while preserving or redirecting adaptive immune components and antimicrobial defenses. This coordinated, multi-faceted action likely underlies the restoration of immune homeostasis and contributes to the alleviation of *Pneumocystis*-induced pulmonary pathology, as evidenced by the improved histological outcomes.

T lymphocytes, particularly CD4+ T cells, play a central role in controlling *Pneumocystis* infection (Hanano and Kaufmann, 1998; Eddens and Kolls, 2015). Depletion of CD4+ T cells leads to impaired immune responses, elevated pathogen burden, and severe lung damage (Beck et al., 1991; Beck et al., 1996). Our findings revealed that Ba treatment restored CD4+ T cell counts, reduced *Pneumocystis* burden, and alleviated lung injury. Additionally, Ba′s effects on oxidative stress parameters, including increased GSH-Px, SOD, and T-AOC levels, as well as decreased MDA concentrations, highlight its antioxidative properties. These results suggest that Ba not only restores immune function but also protects against oxidative damage caused by *Pneumocystis* infection.

Integrated analysis of the host-directed GO, KEGG, and GSEA results suggest coordinated activation in the “immune-inflammation-oxidative stress” network following PCP infection. This network comprises interconnected immune, inflammatory, and oxidative stress pathways that may collectively contribute to PCP pathogenesis and lung injury. Following Ba intervention, characteristic shifts were observed in the key pathways within this network.

First, the enrichment of the Toll-like receptor (TLR) signaling pathway, including the regulation of TLR4 and TLR2 signaling, aligned closely with our experimental validation of TLR2 and TLR4 expression across groups. TLR2 and TLR4 serve as critical upstream sensors within the TLR pathway, mediating the activation of downstream inflammatory cascades in response to pathogenic stimuli (Wang et al., 2024; Pandey et al., 2025; Crammond et al., 2026). Notably, experimental results demonstrated that Ba treatment significantly decreased TLR2 and TLR4 expression. Given that TLR2/4 signaling is a major driver of excessive inflammatory responses during infection (Le et al., 2023; Wang et al., 2024; Pandey et al., 2025; Crammond et al., 2026), these findings suggest that Ba may exert its anti-PCP effect by suppressing TLR2/4–mediated inflammatory activation. This interpretation is consistent with the well-established role of Ba as an inhibitor of TLR2/4 expression and downstream signaling in various inflammatory disease models, including LPS–induced inflammation, periodontitis, cerebral ischemia, and fever (Tu et al., 2011; Jiang et al., 2020; Fu et al., 2021). Second, concerning inflammatory amplification, the downregulation of pathways including regulation of inflammatory response, Chemokine signaling pathway, Chemokine-mediated signaling pathway, Chemokine activity, NF-κB signaling pathway, Cytokine-cytokine receptor interaction, and Cytokine–mediated signaling pathway, corroborates previous reports that Ba treatment attenuates the expression of inflammatory mediators such as CCL2, CCL9, CXCR3, CXCL9, CXCL10, CXCL11, MMP2, and MMP9 (Xue et al., 2025). This consistency further supports the anti–inflammatory role of Ba during PCP infection. Notably, the NF-κB signaling pathway, a central hub linking TLR4 activation to cytokine production, appears to be synergistically inhibited through the suppression of upstream TLR2/4 signaling (Xu et al., 2022; Le et al., 2023; Wang et al., 2024; Nie et al., 2025; Crammond et al., 2026), collectively contributing to the therapeutic mechanism of Ba against PCP.

Regarding adaptive immune response, significant enrichment was observed in pathways related to T cell regulation, including regulation of T cell activation, T cell differentiation involved in immune response, Th17 cell differentiation, Th 17 type immune response, T cell receptor signaling pathway, Lymphocyte activation involved in immune response, as well as antigen processing and presentation and PD-1/PD-L1 checkpoint signaling. These bioinformatic predictions are consistent with our prior flow cytometry results showing that Ba–treated animals exhibited significantly increased CD4^+^ T cell counts and decreased CD8^+^ T cell counts, indicating that Ba modulates T–cell responses during PCP infection (Xue et al., 2025). Moreover, pathways associated with oxidative stress, such as response to oxidative stress, reaction oxygen species metabolic process, and Ferroptosis, along with alterations in oxidative stress biomarkers (elevated SOD, GSH-Px, and T-AOC; reduced MDA) (Xue et al., 2025) and the expression of Nrf2 observed in this study, collectively suggest that Ba treatment significantly enhances antioxidative defense mechanisms. In addition, the enrichment of pathways involved in antigen processing and presentation (antigen processing and presentation, antigen processing and presentation of peptide antigen), lysosomal function (Lysosome), macrophage activation (macrophage activation), and phagosome formation, combined with pathogen-oriented transcriptomic findings, aligns with previously reported reductions in *Pneumocystis* burden following Ba treatment (Xue et al., 2025). This supports the possibility that Ba exerts direct or indirect antimicrobial effects that may contribute to pathogen clearance. In summary, the findings from Dual RNA-Seq analysis and the experimental validation presented in this study are complementary and mutually supportive, implying that baicalin may exert its anti-PCP effects through coordinated modulation of the immune-inflammation-oxidative stress network associated with PCP pathogenesis.

### 4.2 IDO1 as a host candidate target for Ba

IDO1, an immunoregulatory enzyme that metabolizes tryptophan into kynurenine, has been implicated in the pathogenesis of various inflammatory conditions, including chronic infections and autoimmune diseases (Yeung et al., 2015; Li et al., 2017; Guo et al., 2019). Elevated IDO1 expression in lung tissues of PCP-untreated rats correlated positively with *Pneumocystis* burden and pulmonary injury, consistent with its role in promoting inflammation and oxidative stress through excessive ROS production (Gao et al., 2021; Salminen, 2022; Gao et al., 2024). After Ba treatment, IDO1 expression levels were significantly reduced, and oxidative stress biomarkers improved, suggesting that Ba mitigates PCP-induced oxidative damage by downregulating IDO1 expression. These findings support further investigation of IDO1 as a potential therapeutic target for PCP.

### 4.3 Potential activation of Nrf2 signaling by Ba

The Nrf2 signaling pathway is a critical regulator of antioxidant gene expression and plays a protective role against inflammation-induced lung damage (Kobayashi et al., 2016; Liu et al., 2019; Barnes, 2020; Cuadrado et al., 2020; Saha et al., 2020; Liu et al., 2021). Activation of Nrf2 has been linked to reduced pro-inflammatory cytokine levels, including IL-6 and IL-1β, and improved recovery from respiratory disorders such as COPD and pulmonary fibrosis (Kobayashi et al., 2016; Liu et al., 2019). In our study, Ba treatment enhanced Nrf2 expression and upregulated the downstream antioxidant enzyme HO–1, suppressed inflammatory cytokines, and alleviated oxidative stress, thus supporting the role of Nrf2 potential activation in Ba′s anti–PCP effects. Moreover, these findings align with previous studies demonstrating the anti-inflammatory and antioxidant properties of natural compounds, including flavonoids, in pulmonary diseases (Guan et al., 2013; Cheng et al., 2015; Jiao et al., 2017; Cho et al., 2019; Kubo et al., 2019; Boots et al., 2020; Lian et al., 2020; Islam et al., 2021; Yang et al., 2021; Liu et al., 2022; Xue et al., 2025).

### 4.4 *PcRtt109*: a key pathogen-targeted mechanism of Ba

*PcRtt109*, a fungal–specific histone acetyltransferase (HAT), is essential for DNA replication, repair, and fungal virulence (Kottom et al., 2011). Recent studies have identified *PcRtt109* as a promising therapeutic target against *Pneumocystis* (Dahlin et al., 2014). Ba treatment was associated with reduced *PcRtt109* transcript abundance and favorable molecular docking predictions, suggesting that *PcRtt109* may represent a pathogen-associated candidate target involved in the anti-PCP activity of Ba. Downregulation of *PcRtt109* was associated with disrupted nucleotide biosynthesis and inhibited cell cycle progression in *Pneumocystis*, consistent with its proposed role as a regulator of fungal development and virulence (Driscoll et al., 2007; Wurtele et al., 2012; Zhang et al., 2021). Furthermore, molecular docking analysis and the molecular dynamics simulations indicated that Ba exhibits a high binding affinity and stable binding pose for *PcRtt109*, lending computational support to the possibility of an interaction between Ba and this fungal enzyme. In addition to *PcRtt109*, Ba appears to modulate expression of multiple other pathogen genes involved in protein degradation, cell cycle regulation, metabolism, and surface interactions, highlighting a broad-spectrum pathogen-targeted effect.

Our previous study demonstrated that Ba alleviates *Pneumocystis* infection load, as evidenced by both etiological staining and qPCR (Xue et al., 2025). In the present study, Ba treatment was associated with a concentration-dependent decrease in *PcRtt109* expression. Together, these observations suggest that *PcRtt109* may represent a treatment-responsive gene associated with the antifungal effects of Ba, although its precise functional role remains to be established.

### 4.5 Pathogen-targeted mechanisms of Ba in *Pneumocystis*

In addition to modulating host responses, dual RNA-Seq analysis revealed that Ba exerts significant regulatory effects on *Pneumocystis* at the transcriptional level, highlighting a multifaceted pathogen–targeted mechanism. Notably, several key genes involved in proteostasis, cell cycle regulation, metabolism, and host–pathogen interaction exhibited differential expression patterns in response to Ba treatment (Skalski et al., 2015; Williams and Rousseau, 2022).

Proteasome-related genes such as 26S proteasome regulatory subunit Rpn12 and E3 ubiquitin-protein ligase Ptr1 were upregulated following Ba intervention, contrasting their downregulation during active infection. This suggests that Ba may disrupt *Pneumocystis* protein homeostasis by modulating the ubiquitin-proteasome system, which is crucial for degradation of misfolded proteins and regulation of cell cycle progression in this obligate pathogen. The upregulation of proteasome subunits could reflect enhanced protein turnover or a stress response leading to impaired pathogen proliferation. Similar patterns in other pathogenic fungi have been associated with adaptive responses to environmental challenges or antimicrobial agents, suggesting a conserved mechanism of coping with cellular stress (Smalle and Vierstra, 2004; Finley et al., 2012; Yang et al., 2022; Jang et al., 2024).

Genes implicated in cell division and septation, including Septation protein 7 and Anaphase-promoting complex subunit 10, also exhibited increased expression upon Ba exposure but were suppressed under infection conditions. Given that *Pneumocystis* relies on tightly regulated cell cycle machinery for replication within the host lung environment, these expression changes likely indicate Ba-induced perturbation of pathogen growth dynamics, dysregulation of these processes may interfere with the proliferative capacity and life cycle progression of *Pneumocystis*, ultimately impeding its ability to sustain infection. These expression changes likely indicate Ba–induced perturbation of pathogen growth dynamics (Krapp et al., 2006; Krapp and Simanis, 2008; Barford, 2011b; Barford, 2011a; Ostapenko and Solomon, 2011; Dey and Pollard, 2018; Thorn et al., 2024).

Altered expression of metabolic pathway-related genes, such as cystathionine beta-synthase and amino acid transport system proteins, indicates that Ba disrupts essential biosynthetic and nutrient acquisition mechanisms. Since *Pneumocystis* lacks several *de novo* amino acid synthesis pathways and relies heavily on scavenging from the host, the perturbation of amino acid metabolism could severely impact its viability (Merali and Clarkson, 2004; Kutty et al., 2008; Perez-Leal et al., 2011; Ma et al., 2016).

Moreover, surface glycoproteins, particularly major surface glycoprotein 2 (MSG2) and associated domain-containing proteins, as well as membrane remodeling factors, showed significant downregulation in the Ba–treated group compared to the *Pneumocystis* pneumonia model group. These surface glycoproteins play critical roles in pathogen adherence to host cells, immune evasion, and host-pathogen interactions in *Pneumocystis*, suggesting that Ba may weaken *Pneumocystis*’s ability to colonize host tissues, evade immune surveillance, and persist in the host lung by altering its surface architecture (Kutty et al., 2001; Ma et al., 2018; Kottom et al., 2020; Ma et al., 2020; Kottom et al., 2022).

Conversely, genes such as pre-mRNA-splicing factor ATP-dependent RNA helicase PRP43, asparagine-tRNA ligase, and chromatin remodeling factor mit1, which were elevated during infection, were markedly suppressed by Ba intervention, reflecting a broad inhibitory effect of Ba on critical transcriptional and post-transcriptional regulatory pathways required for normal pathogen function (Perez-Martin, 1999; Martin et al., 2002; Creamer et al., 2014; Bohnsack et al., 2022; Lewis et al., 2024).

The observed reduction in *Pneumocystis* burden following Ba treatment in our previous study (Xue et al., 2025) is supported by pathogen-focused transcriptomic analysis, which reveals a concerted suppression of the organism’s core metabolic and proliferative pathways. Ba likely induces a state of “metabolic arrest” in *Pneumocystis*, characterized by significant downregulation of glycolysis, pentose phosphate pathway, and purine metabolism, thereby limiting energy production and biosynthetic capacity essential for replication. Concurrent inhibition of cell cycle progression and DNA replication may explain the halted pathogen expansion observed *in vivo*. These transcriptional changes collectively depict a pathogen under profound bioenergetic and replicative stress, effectively explaining the decrease in organism load. This multi-pathway targeting strategy suggests a potential advantage in mitigating resistance development. Our findings position Ba as a promising candidate for further exploration as an adjunctive therapy, potentially acting synergistically with standard treatments to target *Pneumocystis* through complementary mechanisms.

Collectively, these findings suggest that Ba targets *Pneumocystis* through multi–target modulation of pathways and multiple biological processes–disrupting protein homeostasis, interfering with cell cycle regulation, impairing metabolic adaptation, and weakening host interaction mechanisms.

This multi-target, multi-pathway modulation supports the therapeutic potential of Ba in combating *Pneumocystis* infection, such as impairing pathogen viability and pathogenicity, complementing its roles in host immune modulation, underscoring Ba′s comprehensive therapeutic potential against PCP via dual modulation of both host and pathogen molecular networks, and providing a mechanistic basis for its inclusion in anti–PCP strategies.

### 4.6 Multi-target and multi-pathway therapeutic potential of Ba

PPI network analysis revealed complex interactions among the 14 key proteins identified in this study, including Nrf2, IDO1, TLR signaling mediators TLR2/4, matrix metalloproteinases (MMP2, MMP9), and oxidative stress markers (SOD, GSH-Px). These findings suggest that Ba exerts therapeutic effects against PCP by modulating multi-target and multi-pathway processes, including suppressing inflammation, oxidative stress, antifungal defense, and protease-antiprotease imbalance. Such multifunctional therapeutic properties support the promise of Ba as a natural compound with significant clinical utility.

Furthermore, this study employed an efficient and precise computational approach, integrating molecular docking and molecular dynamics simulations (Wu et al., 2022; Sahu et al., 2024; Sur and Nimesh, 2025; Wang et al., 2025), to provide molecular-level evidence for the multi-target effects of Ba from a structural biology perspective. Molecular docking validated strong binding affinity of Ba towards core candidate targets, including host proteins Nrf2, IDO1, TLR2, and TLR4, as well as the pathogen-derived protein *PcRtt109*. Subsequently, molecular dynamics simulations revealed that these ligand–target complexes remained highly stable throughout the trajectories. Both techniques consistently indicated that Ba exhibits the strongest binding affinity for *PcRtt109*, superior to the other four target proteins. This unique dual-targeting strategy, concurrently acting on pivotal host and pathogen targets, provides molecular-level insights into the cooperative therapeutic potential of Ba.

Importantly, our pathogen-centered transcriptome analysis further demonstrated that Ba simultaneously influences multiple critical biological pathways in *Pneumocystis*, including those related to protein homeostasis, metabolism, cell division, and surface adherence. These pathogen–targeted effects, combined with the host-directed modulation described above, underscore Ba′s dual–action profile. This comprehensive multi-target and multi-pathway approach likely contributes to the robust efficacy of Ba against PCP by impairing both pathogen fitness and host-pathogen interactions. Furthermore, such a multifaceted mode of action and multifunctional therapeutic properties may reduce the risk of resistance development, underscoring the promise of Ba as a natural compound with significant clinical utility for both prevention and treatment of PCP.

### 4.7 Safety considerations of Ba intervention

Alongside its efficacy, the safety profile of Ba within the therapeutic context is a crucial consideration. In the present study, the findings corroborate the favorable safety profile of Ba within the dose range employed. Pharmacodynamically, no statistically significant differences were observed between the Ba-control group (healthy animals receiving Ba intervention alone) and the vehicle control group across key endpoints, including pulmonary cytokine levels, histopathological features, and *Pneumocystis* burden. This indicates that Ba administration, in the absence of infection, did not induce detectable lung injury or nonspecific inflammatory responses. Furthermore, this local safety assessment aligns with systemic evidence from our prior research (Xue et al., 2025), in which identical dosing of Ba resulted in no pathological alterations or inflammatory infiltration in cardiac tissue, supporting its good tolerability at the organ level. Taken together, under the specific dosage and treatment duration applied in this study, Ba demonstrated no measurable toxicity or adverse effects in uninfected animals, thereby providing a critical safety context for its observed anti-infective efficacy in the PCP model. It is important to note that drug safety is a multidimensional parameter. The conclusions herein are derived from a specific animal model, dosing regimen, and set of observational indicators. Further studies involving extended treatment periods, varied dose gradients, and translational clinical evaluation are warranted to comprehensively characterize its safety profile.

### 4.8 Limitation and future perspective

This study has multiple limitations. First, the transcriptomic differences observed between the immunocompetent control and the PCP group are inevitably confounded by the immunological effects of dexamethasone-induced immunosuppression. In the absence of a non-infected immunosuppressed control group, the observed differential expression, likely reflects a combination of responses to both immunosuppression and *Pneumocystis* infection, limiting the attribution of individual transcriptomic changes specifically to the pathogen. Second, the sample size is relatively small, with three biological replicates per group. While this sample size is commonly used in exploratory transcriptomic studies, it may have reduced the statistical power to detect genes with modest expression changes. Future studies incorporating larger cohorts will help strengthen the robustness of these findings. Third, experimental validation was limited to selected candidate targets. Although dual RNA-seq identified five key targets, numerous additional significant DEGs remain to be functionally characterized. With uncertain implications. Further investigation of these candidates may provide a more comprehensive understanding of the molecular mechanisms underlying the effects of Ba. Finally, the molecular interactions between Ba and its target proteins were inferred primarily from molecular docking and molecular dynamics simulations. Although these approaches provide structural support for potential ligand-target interactions, direct experimental validation is needed. Future studies should evaluate the specific binding between Ba and candidate targets using biophysical approaches such as surface plasmon resonance or isothermal titration calorimetry, together with functional assays examining Nrf2 nuclear translocation, IDO1 enzymatic activity, kynurenine production, and other downstream signaling events. Such studies would further clarify the molecular mechanisms underlying the therapeutic effects of Ba against PCP.

## 5 Conclusion

In conclusion, our study provides important insights into the molecular mechanisms underlying Ba′s therapeutic effects against PCP by integrating host and pathogen transcriptome analyses. These analyses suggest that Ba modulates both host and pathogen molecular pathways, and is associated with reduced inflammatory and oxidative stress responses, altered host immune signaling, and changes in the expression of pathogen genes implicated in virulence and cellular functions in *Pneumocystis*-infected immunosuppressed rats. In-depth analysis of *Pneumocystis* transcriptome data further suggests that Ba may exerts multi-target effects that disrupt essential biological processes within the pathogen, complementing its immunomodulatory activity in the host. These findings deepen our knowledge of Ba′s mode of action, and provide a foundation for future studies of its therapeutic potential for PCP. Additionally, this research underscores the value of dual RNA-Seq in elucidating host-pathogen interactions and facilitating the development of traditional Chinese medicine-based therapeutics.

## Data Availability

The datasets presented in this study can be found in online repositories. The raw sequencing reads have been deposited in the NCBI Sequence Read Archive (SRA) under BioProject accession PRJNA1111249 and SRA accession SRR29014119–SRR29014127.

## Footnotes

1
http://asia.ensembl.org/Rattus_norvegicus/Info/Index?db=core,mRatBN7.2

2
https://fungidb.org/fungidb/app/record/dataset/NCBITAXON_1408658,FungiDB-67_PcariniiB80

3
https://bioinformatics.psb.ugent.be/webtools/Venn/

4
https://bioinfogp.cnb.csic.es/tools/venny/index.html

5
https://cn.string-db.org/

6
https://pubchem.ncbi.nlm.nih.gov/

7
https://www.uniprot.org/

## Glossary

ANOVA: one-way analysis of variance
Ba: Baicalin
BP: Biological Process
CCL: Chemokine (C-C motif) ligand
CD4: Cluster of Differentiation 4
COPD: chronic obstructive pulmonary disease
CXCL: C-X-C motif chemokine ligand
CXCR: chemokine C-X-C-motif receptor
DEGs: differentially expressed genes
*dhps*: dihydropteroate synthase
ELISA: enzyme-linked immunosorbent assay
GSEA: Gene Set Enrichment Analysis
GSH-Px: Glutathione peroxidase
GO: gene ontology
HAART: highly active antiretroviral therapy
HAT: histone acetyltransferase
HIV: human immunodeficiency virus
HO-1: heme oxygenase-1
IDO1: indoleamine 2,3-dioxygenase 1
IL: Interleukin
IHC: immunohistochemistry
KEGG: kyoto encyclopedia of genes and genomes
MCP: monocytic chemotactic protein
MDA: malondialdehyde
MMP: matrix metalloproteinases
MMPBSA: Molecular Mechanics/Poisson-Boltzmann Surface Area
MSG: major surface glycoprotein
NF-κB: nuclear factor-κB
NLRs: Nucleotide-binding Oligomerization Domain-like Receptors
NOD: Nucleotide-binding Oligomerization Domain
Nrf2: nuclear factor erythroid 2-related factor 2
*Pc*: *Pneumocystis carinii*
PCP: *Pneumocystis* pneumonia
*PcRtt109*: *Pneumocystis Rtt109*
PCR: polymerase chain reaction
PPI: Protein–protein interaction
Rg: radius of gyration
RMSD: root mean square deviation
RMSF: root mean square fluctuation
RNA-Seq: RNA sequencing
ROS: reactive oxygen species
RT-qPCR: quantitative real-time PCR
SASA: solvent-accessible surface area
SD: standard deviation
Seq: sequence
SOD: Superoxide dismutase
SRA: Sequence Read Archive
JAK-STAT: Janus kinase-signal transducer and activator of transcription
T-AOC: total antioxidant capacity
Th17: T helper cell 17
TLR: toll-like receptor
TMP-SMX: Trimethoprim-sulfamethoxazole
TNF: Tumor necrosis factor
3D: three-dimensional
