# Supplementary material for: Integrated dual transcriptome sequencing and experimental validation reveal potential mechanisms of baicalin against pneumocystis pneumonia in immunosuppressed rats
Source: Front Microbiol. 2026 Aug 26;17:1923465. doi: 10.3389/fmicb.2026.1923465 (PMC13553826; doi:10.3389/fmicb.2026.1923465)
Supplement: Supplementary file 1 [file Data_sheet_1.zip › Supplementary Materials/Supplementary Material 4/C.Venn Diagram of Overlapping Targets.pdf]

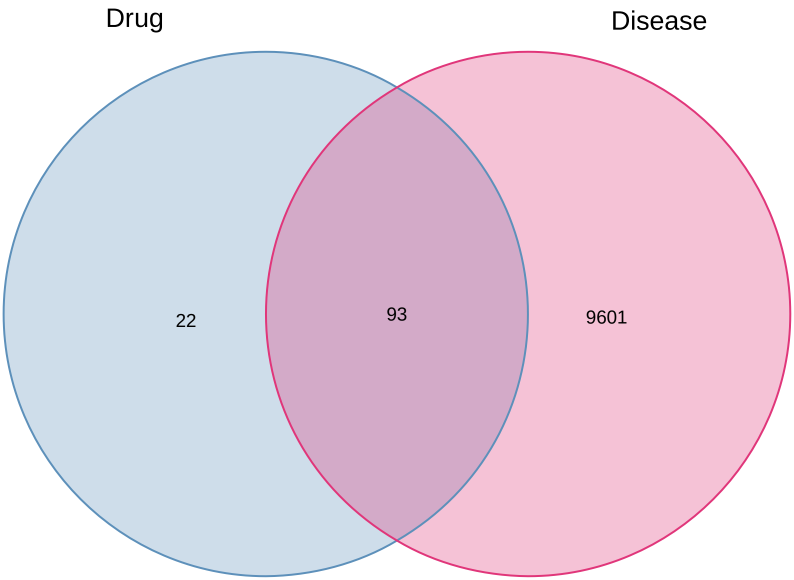
Drug
Disease
22
93
9601
