# Supplementary material for: Integrated dual transcriptome sequencing and experimental validation reveal potential mechanisms of baicalin against pneumocystis pneumonia in immunosuppressed rats
Source: Front Microbiol. 2026 Aug 26;17:1923465. doi: 10.3389/fmicb.2026.1923465 (PMC13553826; doi:10.3389/fmicb.2026.1923465)
Supplement: Supplementary file 1 [file Data_sheet_1.zip › Supplementary Materials/Supplementary Material 7/B.Coordinate mapping of grid boxes.docx]

Coordinate mapping of grid boxes across selected protein targets

| Target name | Size measurement of grid box | |
| --- | --- | --- |
|  | center | dimensions |
| Nrf2 | x = -1, y = -2.2, z = -4.6 | x = 93.8, y = 65.2, z = 100.1 |
| IDO1 | x = -7.9, y = 1.6, z = -0.7 | x = 57.1, y = 45.2, z = 64 |
| TLR2 | x = -3.3, y= 89.1, z = 16.5 | x = 64, y = 55.9, z = 48.8 |
| TLR4 | x = 16.1, y = -4.5, z = 29 | x = 49.3, y = 49.8, z = 41.5 |
| PcRtt109 | x = -6.9, y = 2.5, z = 1.5 | x = 75.4, y = 51.6, z = 53.7 |
