# Supplementary material for: Integrated dual transcriptome sequencing and experimental validation reveal potential mechanisms of baicalin against pneumocystis pneumonia in immunosuppressed rats
Source: Front Microbiol. 2026 Aug 26;17:1923465. doi: 10.3389/fmicb.2026.1923465 (PMC13553826; doi:10.3389/fmicb.2026.1923465)
Supplement: Supplementary file 1 [file Data_sheet_1.zip › Supplementary Materials/Supplementary Material 7/C.Binding sites.docx]

Binding sites between Ba and the candidate target proteins

Nrf2_baicalin: Try46, Glu47, Gly529, Asp533.

IDO1_baicalin: Phe231, His232, Arg235, Pro245, Asn244, Lys246.

TLR2_baicalin: Thr64, His62, Ser61, Ile58, Asn94, Arg88.

TLR4_baicalin: His42, Ser47, Lys21, Tyr20.

PcRtt109_baicalin: Lys143, His144, Ser85, Asp84, Leu146. Ala83, Trp154.
